# Chronic obstructive pulmonary disease affects outcome in surgical patients with perioperative organ injury: a retrospective cohort study in Germany

**DOI:** 10.1186/s12931-024-02882-3

**Published:** 2024-06-20

**Authors:** Nadine Hochhausen, Mare Mechelinck, Andreas Kroh, Rolf Rossaint, Felix Kork

**Affiliations:** 1 Department of Anesthesiology, Medical Faculty, RWTH Aachen University, Pauwelsstrasse 30, 52074 Aachen, Germany; 2 Department of General, Visceral, Pediatric, and Transplantation Surgery, Medical Faculty, RWTH Aachen University, Aachen, Germany

**Keywords:** Chronic obstructive pulmonary disease, Perioperative organ injury, In-hospital mortality

## Abstract

**Background:**

The impact of chronic obstructive pulmonary disease (COPD) on outcome in perioperative organ injury (POI) has not yet been investigated sufficiently.

**Methods:**

This retrospective cohort study analysed data of surgical patients with POI, namely delirium, stroke, acute myocardial infarction, acute respiratory distress syndrome, acute liver injury (ALI), or acute kidney injury (AKI), in Germany between 2015 and 2019. We compared in-hospital mortality, hospital length of stay (HLOS) and perioperative ventilation time (VT) in patients with and without COPD.

**Results:**

We analysed the data of 1,642,377 surgical cases with POI of which 10.8% suffered from COPD. In-hospital mortality was higher (20.6% vs. 15.8%, *p* < 0.001) and HLOS (21 days (IQR, 12–34) vs. 16 days (IQR, 10–28), *p* < 0.001) and VT (199 h (IQR, 43–547) vs. 125 h (IQR, 32–379), *p* < 0.001) were longer in COPD patients. Within the POI examined, AKI was the most common POI (57.8%), whereas ALI was associated with the highest mortality (54.2%). Regression analysis revealed that COPD was associated with a slightly higher risk of in-hospital mortality (OR, 1.19; 95% CI:1.18–1.21) in patients with any POI.

**Conclusions:**

COPD in patients with POI is associated with higher mortality, longer HLOS and longer VT. Especially patients suffering from ALI are susceptible to the detrimental effects of COPD on adverse outcome.

**Supplementary Information:**

The online version contains supplementary material available at 10.1186/s12931-024-02882-3.

## Background

Perioperative organ injury is a serious complication after surgical procedures and associated with high morbidity and mortality [1]. Perioperative organ injury or failure results from insufficient organ perfusion and surgical affection leading to inflammation and ischemia [2]. All organs can be affected resulting in severe complications like respiratory failure, acute kidney injury, neurological as well as cardiovascular complications [2]. Various attempts were made to determine patients at risk of perioperative complications including screening methods, scores, or laboratory parameters [3–9]. Despite valuable findings in identifying potential risk factors, a high mortality of perioperative complications continues to be observed. The Lancet Commission on Global Surgery identified that postoperative death account for the third largest causes of death [10]. Moreover, findings show a postoperative mortality of 4% before hospital discharge [11]. Besides perioperative organ injury, demographic change, more complex operations, and increasingly ill patients suffering from chronic diseases are some of the reasons for perioperative mortality [12].

One of the most common chronic diseases is chronic obstructive pulmonary disease (COPD). COPD is characterised by respiratory symptoms based on airflow obstruction [13, 14] and is one of the most common causes of death worldwide [10]. The number of patients with COPD will continue to rise due to the aging population and continuous exposure to risk factors [14, 15]. It is already known that COPD is associated with adverse outcome after various surgical procedures [16–20]. However, it has not yet been investigated in detail whether COPD patients suffering from pathological perioperative organ injury, like delirium, stroke, acute myocardial infarction (AMI), acute respiratory distress syndrome (ARDS), acute liver injury (ALI) and acute kidney injury (AKI), are more likely to develop an adverse outcome compared to patients without COPD. Moreover, we did not find any data reporting the impact of COPD on outcomes in patients with different types of perioperative organ injury. We hypothesised that COPD deteriorates the outcomes of surgical patients already suffering from perioperative organ injury.

Therefore, we conducted a population-based retrospective cohort study in hospitalised surgical patients with perioperative organ injury. We sought to estimate the impact of COPD on in-hospital mortality, hospital length of stay (HLOS) and perioperative ventilation time (VT) in these patients.

## Methods

### Patients and data source

No Institutional or Review Board approval was required since de-identified data was used. All data was accessed via controlled remote data processing without access to the actual data.

We prepared an analysis protocol as a Stata do-file (Stata BE 17 for Windows, StataCorp, College Station, TX, USA) and tested it on the data structure files provided by the Federal Statistical Office. Afterwards, the analysis protocol was sent to the Federal Statistical Office to perform it on the original data (Stata 15 for Windows, StataCorp, College Station, TX, USA). The de-identified results were made available to the authors.

In this retrospective cohort study, we analysed data from the German Diagnoses Related Groups (G-DRG) Statistik (Source: RDC of the Federal Statistical Office and Statistical Offices of the Federal States, Source DOI: 10.21242/23141.2019.00.00.1.1.1, 10.21242/23141.2018.00.00.1.1.0, 10.21242/23141.2017.00.00.1.1.0, 10.21242/23141.2016.00.00.1.1.0, 10.21242/23141.2015.00.00.1.1.0, own calculations), provided by the Federal Statistical Office (Statistisches Bundesamt, www.destatis.de).

In Germany, hospitals are reimbursed on a hospital-case basis using the G-DRG-system. Reimbursement for the DRGs is based on differences in treatment costs referred to, e.g., comorbidities present during hospitalisation. Therefore, hospitals must provide various data, such as sociodemographic data, main and side diagnoses, coded in the International Classification of Diseases and Related Health Problems, tenth edition, German Modification (ICD-10-GM). In addition, surgical and other procedures, coded in the Operationen- und Prozedurenschlüssel (OPS), the German version of the International Classification of Procedures in Medicine (ICPM) as established by the World Health Organization (WHO), must be provided. All data are de-identified and transmitted to the Federal Statistical Office. The Federal Statistical Office offers data structure files with the same syntax and coding as the original data for scientific use. However, the data are shuffled and therefore without sense.

### Inclusion and exclusion criteria

First, we included all patients undergoing any type of surgery, regardless of the indication, in Germany between January 1st, 2015, and December 31st, 2019. Then, we excluded patients with age under 18 years and patients with prior lung or heart and lung transplantation. In addition, we excluded surgical cases without perioperative organ injury.

Consequently, our study population consisted of surgical patients suffering from perioperative organ injury. Each hospitalisation is considered as one case.

### Variables

Within the G-DRG Statistik, all surgical cases between 2015 and 2019 were accessible. Surgical cases were set by the Federal Statistical Office as a minimum of one procedure code from Chap. 5 of the OPS (Additional File 1). In addition, access to ICD-10-GM coded diagnoses, such as COPD, were provided. New variables were created by using ICD-10-GM diagnosis codes (Additional File 2). Perioperative organ injury was defined and coded as delirium, stroke, AMI, ARDS, ALI, and AKI. We chose these endpoints because they are well defined and easily extractable from the database due to coding. In addition, we calculated the Charlson Comorbidity Index (CCI) with its items as published by Quan et al. [21]. Quan et al. assigned ICD codes to various comorbidities also differentiating between, e.g., mild, and moderate to severe liver disease.

### Outcome

The primary endpoint was in-hospital mortality. Secondary endpoints were HLOS and VT, which are original variables within the G-DRG Statistik.

### Confounders

As confounders with the potential to affect the endpoints, we considered age, sex, and patients´ comorbidities. The comorbidities were evaluated using the items of the Charlson Comorbidity Index (CCI) [22]. The CCI items were abstracted from the ICD-10-GM as described by Quan [21]. In addition, we considered systemic inflammatory response syndrome (SIRS)/ sepsis and pulmonary embolism as possible confounders. These variables were created by using ICD-10-GM diagnosis codes (Additional File 2).

### Statistical analysis

No sample size or power considerations were made due to the nature of a retrospective cohort study. The G-DRG Statistik includes a complete census of German hospital data, so that no missing data occur. The analysis protocol was coded by the authors and analysed by the Federal Statistical Office (Stata 15 for Windows, StataCorp, College Station, TX, USA). Statistical significance was considered for *p* < 0.01.

We reported frequencies as numbers and percentages, continuous variables as median and inter-quartile ranges (IQR). Continuous variables were compared using the Mann-Whitney-U-test, categorical variables were compared using the chi-squared-test. Binary logistic regression models were applied to estimate the association between COPD and in-hospital mortality, when suffering from any or different types of perioperative organ injury. In addition, robust regression models were applied to estimate the association between COPD and HLOS as well as between COPD and VT, when suffering from any or different types of perioperative organ injury. We introduced dependent variables in the regression models based on clinical relevance and availability in the database. We fitted and cross-validated binary logistic regression models using the Stata module cvauroc (k = 10; robustness measure: area under the receiver operating curve (AUROC)) to estimate the association of COPD and in-hospital mortality [23]. Moreover, we fitted and cross-validated robust regression models using the Stata module ‘crossfold’ (k = 10; robustness measure: root mean square error (RMSE)) to estimate the association between COPD, HLOS and VT [24].

The graph was created using GraphPad Prism 8 for Windows (GraphPad, San Diego, CA, USA).

## Results

### Study population

**Fig. 1:**
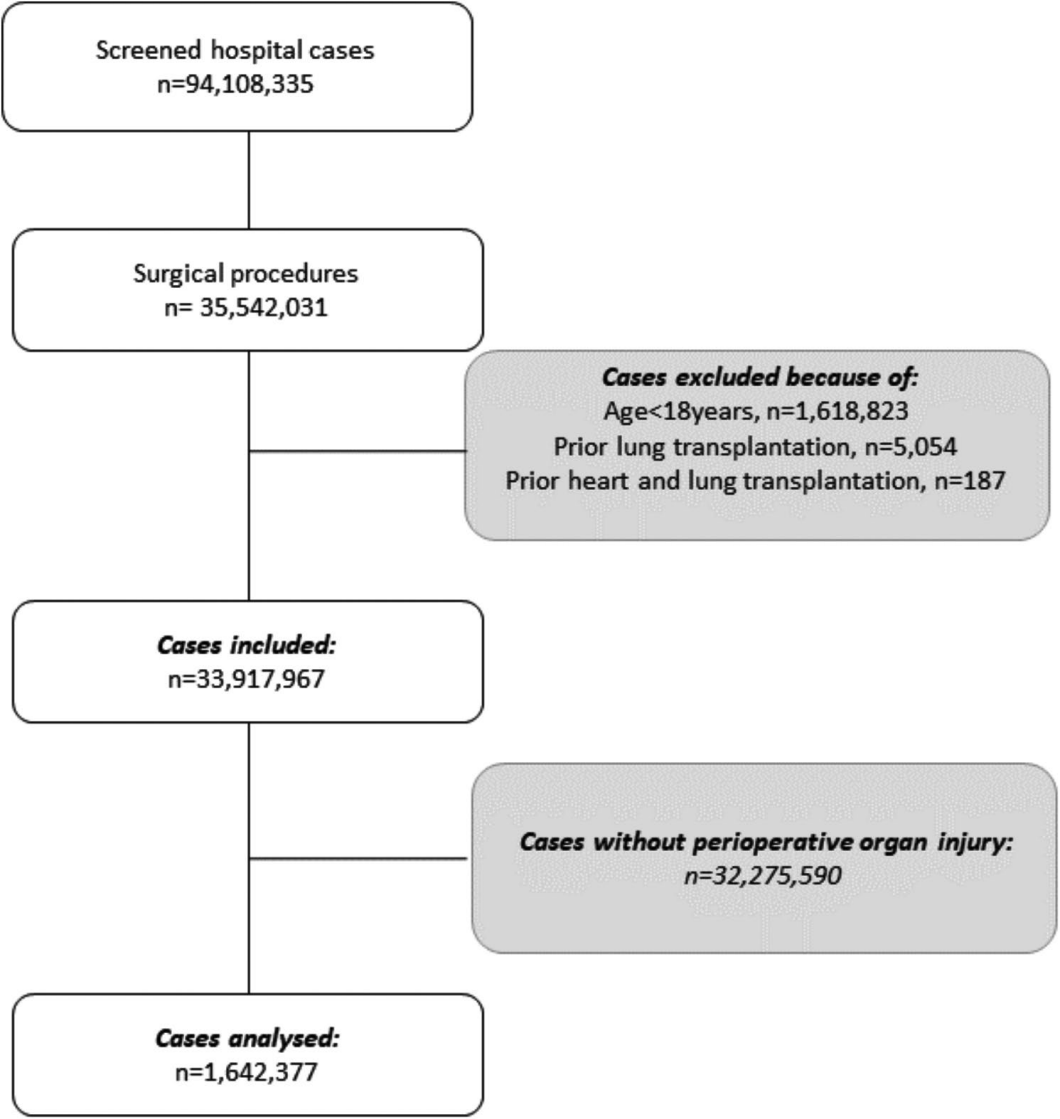
Flow chart of patient inclusion. Flow chart of patient inclusion of a population-based retrospective analysis investigating the impact of COPD on in-hospital mortality, hospital length of stay (HLOS) and ventilation time (VT) in 1,642,377 surgical cases with perioperative organ failure

94,108,335 hospital cases between 2015 and 2019 were screened of which in 35,542,031 cases surgery was performed. Cases with age under 18 years (*n* = 1,618,823), prior lung transplantation (*n* = 5,054) or prior heart-lung transplantation (*n* = 187) were excluded. Of the remaining 33,917,967 surgical cases, 1,339,079 (3.9%) cases were diagnosed with COPD and 1,642,377 cases (4.8%) suffered from any perioperative organ injury according to our definition and were analysed in this study (Fig. 1). Most perioperative organ injuries were observed in operations on the digestive (32.5%) and on the musculoskeletal system (25.3%) (Additional File 3).

Table 1 summarises the characteristics of the study population suffering from perioperative organ injury. The median age of all patients was 76 years (IQR, 66–82), and most of the patients were of male gender (59.0%). The patients suffered most frequently from congestive heart failure (*n* = 569,922; 34.7%), followed by renal disease (*n* = 549,690; 33.5%). The median CCI was 3 (IQR, 2–5).

**Table 1:** Characteristics and outcome of 1,642,377 hospitalised surgical patients with chronic obstructive pulmonary disease (COPD) and without COPD suffering from any perioperative organ injury

Characteristic	All patients (*N* = 1,642,377)	Patients with COPD (*N* = 177,070; 10.8%)	Patients without COPD (*N* = 1,465,307; 89.2%)	*P*-value COPD vs. no-COPD
*Sociodemographic characteristics*				
Median age (IQR)- years	76 (66–82)	75 (67–81)	76 (66–83)	< 0.001
Female- no (%)	674,014 (41.0)	63,549 (35.9)	610,465 (41.7)	< 0.001
Male- no (%)	968,289 (59.0)	113,518 (64.1)	854,771 (58.3)	< 0.001
Unknown- no (%)	74 (0.0)	3 (0.0)	71 (0.0)	< 0.001
Median Charlson comorbidity index (IQR)- pts	3 (2–5)	4 (3–6)	3 (1–5)	< 0.001
*Charlson comorbidity index items- no (%)*				
Myocardial infarction	286,390 (17.4)	34,465 (19.5)	251,925 (17.2)	< 0.001
Congestive heart failure	569,922 (34.7)	89,497 (50.4)	480,425 (32.9)	< 0.001
Peripheral vascular disease	307,305 (18.7)	46,644 (26.3)	260,661 (17.8)	< 0.001
Cerebrovascular disease	347,775 (21.2)	30,900 (17.5)	316,875 (21.6)	< 0.001
Dementia	203,625 (12.4)	16,233 (9.2)	187,392 (12.8)	< 0.001
Chronic pulmonary disease	222,818 (13.6)	177,070 (100.0)	45,748 (3.1)	< 0.001
Rheumatoid disease	28,735 (1.7)	3,844 (2.2)	24,891 (0.02)	< 0.001
Peptic ulcer disease	72,515 (4.4)	8,434 (4.8)	64,081 (4.4)	< 0.001
Liver disease				
Mild	75,728 (4.6)	9,526 (5.4)	66,202 (4.5)	< 0.001
Moderate to severe	39,664 (2.4)	3,246 (1.8)	36,418 (2.5)	< 0.001
Diabetes mellitus				
Uncomplicated	343,469 (20.9)	41,053 (23.2)	302,416 (20.6)	< 0.001
With end-organ damage	180,170 (11.0)	25,361 (14.3)	154,809 (10.6)	< 0.001
Hemiplegia or paraplegia	204,574 (12.5)	16,991 (9.6)	187,583 (12.8)	< 0.001
Renal disease	549,690 (33.5)	73,900 (41.3)	475,790 (32.5)	< 0.001
Cancer				
Non-metastatic	175,990 (10.7)	18,192 (10.3)	157,798 (10.8)	< 0.001
Metastatic	113,875 (6.9)	10,334 (5.8)	103,541 (7.1)	< 0.001
AIDS	1,271 (0.1)	108 (0.06)	1,163 (0.1)	0.009
*Outcome parameter*				
In-hospital mortality- no (%)	268,199 (16.3)	36,396 (20.6)	231,803 (15.8)	< 0.001
Median hospital length of stay- days (IQR)	17 (10–29)	21 (12–34)	16 (10–28)	< 0.001
Median ventilation time- hours (IQR)	135 (33–404)	199 (43–547)	125 (32–379)	< 0.001

no: number, IQR: interquartile range

The in-hospital mortality was 16.3% for patients with any perioperative organ injury, the median HLOS was 17 days (IQR, 10–29) and the median VT was 135 h (IQR, 33–404).

### COPD and any perioperative organ injury

10.8% (*n* = 177,070) of the study population (*n* = 1,642,377) suffered from COPD (Table 1).

The median age in COPD patients was 75 years (IQR, 67–81) and most of the patients were of male gender (64.1%). Patients with COPD were of poorer health condition represented by a higher CCI compared to patients without COPD (4 (IQR, 3–6) vs. 3 (IQR, 1–5), *p* < 0.001). Patients with and without COPD suffered most frequently from congestive heart failure (50.4% vs. 32.9%, *p* < 0.001), followed by renal disease (41.3% vs. 32.5%, *p* < 0.001) (Table 1).

### Impact of COPD on in-hospital mortality, HLOS and VT

COPD patients demonstrated a higher in-hospital mortality compared to patients without COPD (20.6% vs. 15.8%, *p* < 0.001), when suffering from any perioperative organ injury. In addition, median HLOS (21 days (IQR, 12–34) vs. 16 days (IQR, 10–28, *p* < 0.001)) and median VT (199 h (IQR, 43–547) vs. 125 h (IQR, 32–379, *p* < 0.001)) were also higher in patients with COPD compared to patients without COPD (Table 1).

### COPD and different types of perioperative organ injury

In COPD patients with perioperative organ injury, the analysed perioperative organ injuries were observed in the following descending order: AKI (57.8%), delirium (36.5%), AMI (12.8%), stroke (8.5%), ALI (4.3%) and ARDS (3.1%). Within the perioperative organ injuries analysed, patients diagnosed with COPD suffer more often from AKI (57.8% vs. 50.4%, *p* < 0.001), delirium (36.5% vs. 34.6%, *p* < 0.001) and ARDS (3.1% vs. 2.3%, *p* < 0.001) compared to patients without COPD. In contrast, patients without COPD suffered more often from stroke (13.3% vs. 8.5%, *p* < 0.001) and ALI (5.4% vs. 4.3%, *p* < 0.001) (Table 2). AMI was not significantly different between groups (12.8% vs. 13.0%, *p* = 0.088) (Table 2).

**Table 2:** Outcomes of hospitalised surgical patients with chronic obstructive pulmonary disease (COPD) (*n* = 177,070) and without COPD (*n* = 1,465,307) suffering from each type of perioperative organ injury

	COPD	No COPD	*P*-value
Perioperative delirium			
Prevalence- no (%)	64,626 (36.5)	506,901 (34.6)	< 0.001
In-hospital mortality- no (%)	10,021 (15.5)	54,637 (10.8)	< 0.001
Median hospital length of stay- days (IQR)	24 (14–39)	19 (11–31)	< 0.001
Median ventilation time- hours (IQR)	212 (47–589)	121 (33–360)	< 0.001
Perioperative stroke			
Prevalence- no (%)	15,029 (8.5)	194,882 (13.3)	< 0.001
In-hospital mortality- no (%)	2,701 (17.8)	25,683 (13.2)	< 0.001
Median hospital length of stay – days (IQR)	21 (12–36)	16 (10–29)	< 0.001
Median ventilation time- hours (IQR)	291 (74–590)	237 (69–475)	< 0.001
Perioperative acute myocardial infarction			
Prevalence- no (%)	22,736 (12.8)	190,258 (13.0)	0.088
In-hospital mortality- no (%)	4,198 (18.5)	28,615 (15.0)	< 0.001
Median hospital length of stay- days (IQR)	18 (11–30)	14 (9–24)	< 0.001
Median ventilation time- hours (IQR)	121 (25–438)	78 (19–307)	< 0.001
Perioperative acute respiratory distress syndrome			
Prevalence- no (%)	5,540 (3.1)	34,183 (2.3)	< 0.001
In-hospital mortality- no (%)	2,265 (40.9)	15,481 (45.3)	< 0.001
Median hospital length of stay- days (IQR)	30 (18–48)	29 (16–48)	< 0.001
Median ventilation time- hours (IQR)	478 (248–764)	395 (173–685)	< 0.001
Perioperative acute liver injury			
Prevalence- no (%)	7,632 (4.3)	79,853 (5.4)	< 0.001
In-hospital mortality- no (%)	4,137 (54.2)	33,787 (42.3)	< 0.001
Median hospital length of stay- days (IQR)	18 (9–34)	15 (7–29)	< 0.001
Median ventilation time- hours (IQR)	224 (60–549)	162 (47–432)	< 0.001
Perioperative acute kidney injury			
Prevalence- no (%)	102,265 (57.8)	738,160 (50.4)	< 0.001
In-hospital mortality- no (%)	27,054 (26.5)	166,389 (22.5)	< 0.001
Median hospital length of stay- days (IQR)	21 (12–35)	18 (10–31)	< 0.001
Median ventilation time- hours (IQR)	220 (50–566)	147 (39–422)	< 0.001

no: number, IQR: interquartile range

### Impact of COPD on in-hospital mortality, HLOS and VT in patients with different types of perioperative organ injury

**Fig. 2:**
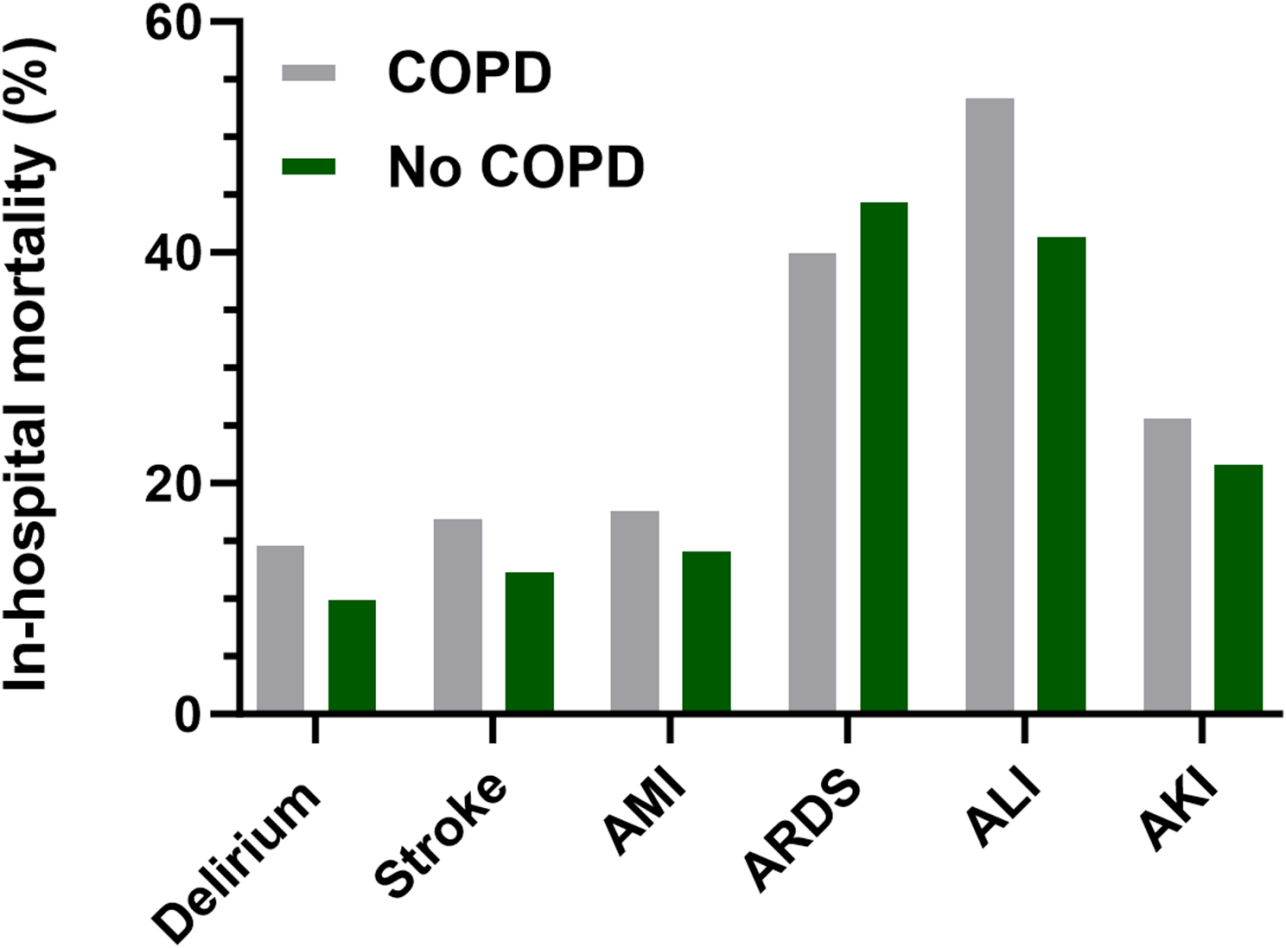
In-hospital mortality of patients with and without COPD. In-hospital mortality of patients with (grey) and without (green) COPD in different types of perioperative organ injuries, namely: delirium, stroke, acute myocardial infarction (AMI), acute respiratory distress syndrome (ARDS), acute liver injury (ALI) and acute kidney injury (AKI)

Patients diagnosed with COPD demonstrated a higher in-hospital mortality compared to patients without COPD in all examined different types of perioperative organ injuries (Table 2; Fig. 2), except in ARDS (40.9% vs. 45.4%, *p* < 0.001). The highest in-hospital mortality was observed in perioperative ALI (54.2%), the lowest in perioperative delirium (15.5%).

Median HLOS was longer in all examined different perioperative organ injuries when suffering from COPD compared to patients without COPD (Table 2). The longest HLOS was observed in ARDS (30 days (IQR, 18–48)), followed by delirium (24 days (IQR, 14–39)), the lowest in perioperative acute myocardial infarction (18 days (IQR, 11–30)) and perioperative acute liver injury (18 days (IQR, 9–34)) (Table 2).

In addition, median VT was also prolonged in COPD patients in all examined different perioperative organ injuries compared to patients without COPD (Table 2). The most prolonged median VT was observed in ARDS (478 h (IQR, 248–764)), followed by perioperative stroke 291 h (IQR, 75–590)). The lowest in perioperative acute myocardial infarction (121 h, (IQR, 25–438) (Table 2).

### Regression model analyses

Table 3 summarises the key findings of regression model analyses. The complete results are available as supplementary material (Additional File 4–24).

**Table 3:** Associations of COPD with mortality, hospital length of stay and perioperative ventilation time in hospitalised surgical patients with perioperative organ injury from different multivariable regression models

	Mortality		Hospital length of stay		Ventilation time	
	**Odds ratio (95% CI)**	**P-value**	**Beta (95% CI)**	**P-value**	**Beta (95% CI)**	**P-value**
COPD in patients with **any perioperative organ injury**	1.19 (1.18–1.21)	< 0.001	2.62 (2.51–2.73)	< 0.001	107.47 (104.01-110.93)	< 0.001
COPD in patients with **delirium**	1.30 (1.27–1.34)	< 0.001	2.80 (2.60–2.99)	< 0.001	143.45 (137.67-149.22)	< 0.001
COPD in patients with **stroke**	1.15 (1.09–1.20)	< 0.001	2.57 (2.14-3.00)	< 0.001	74.04 (62.03–86.05)	< 0.001
COPD in patients with **AMI**	1.10 (1.06–1.15)	< 0.001	2.31 (2.05–2.57)	< 0.001	71.27 (63.57–78.98)	< 0.001
COPD in patients with **ARDS**	0.82 (0.77–0.88)	< 0.001	1.35 (0.51–2.20)	0.002	85.89 (71.03-100.75)	< 0.001
COPD in patients with **ALI**	1.16 (1.10–1.23)	< 0.001	1.25 (0.67–1.83)	< 0.001	82.43 (68.66–96.19)	< 0.001
COPD in patients with **AKI**	1.22 (1.20–1.24)	< 0.001	1.87 (1.72–2.01)	< 0.001	98.69 (94.11-103.26)	< 0.001

AMI: acute myocardial infarction, ARDS: acute respiratory distress syndrome, ALI: acute liver injury, AKI: acute kidney injury

Risk-adjusted associations from twenty-one multivariable regression analyses models in hospitalised surgical patients with perioperative organ injury

#### In-hospital mortality

COPD was associated with a higher odds of in-hospital mortality (odds ratio (OR), 1.19; 95% confidence interval (CI): 1.18–1.21, *p* < 0.001), when suffering from any perioperative organ injury.

For all different perioperative organ injuries examined, COPD was associated with higher odds of in-hospital mortality, except for ARDS (OR, 0.82; 95% CI: 0.77–0.88, *p* < 0.001).

Within the perioperative organ injuries analysed, COPD was associated with the highest risk for in-hospital mortality when suffering from perioperative delirium (OR, 1.30, 95% CI: 1.27–1.34, *p* < 0.001) (Table 3).

#### HLOS

COPD was associated with a longer HLOS (beta, 2.62 days; 95% CI: 2.51–2.73, *p* < 0.001), when suffering from any perioperative organ injury.

Further regression model analyses showed a longer HLOS for all different perioperative organ injuries examined (Table 3). The longest HLOS was observed for COPD, when suffering from perioperative delirium (beta, 2.80 days; 95% CI: 2.60–2.99; *p* < 0.001) (Table 3).

#### VT

In addition, COPD was also associated with a longer VT (beta, 107.47 h, 95% CI: 104.01-110.93, *p* < 0.001), when suffering from any perioperative organ injury.

For all different perioperative organ injuries analysed, COPD was associated with a longer VT, with the longest VT in perioperative delirium (beta, 143.45 h, 95% CI: 137.67-149.22, *p* < 0.001).

#### Confounders

We addressed different confounders in the regression model analysis. The regression model analysis identified SIRS/sepsis (OR, 4.52; 95% CI: 4.47–4.56, *p* < 0.001) and moderate to severe liver disease (OR, 3.80; 95% CI: 3.72–3.89, *p* < 0.001) as the leading risk factors for in-hospital mortality (Additional File 4) when suffering from any perioperative organ injury. SIRS/Sepsis increases the odds for in-hospital mortality by almost 4.5 folds and moderate to severe liver disease by almost 4.0 folds. When considering different types of perioperative organ injury, perioperative liver injury is one of the leading risk factors for in-hospital mortality (Additional File 5–10). Emergency hospital admission was associated with a higher odds of in-hospital mortality (OR 1.23; 95% CI: 1.22–1.24, *p* < 0.001), when suffering from any perioperative organ injury. Within the different perioperative organ injuries examined, emergency hospital admission was associated with a higher odds of in-hospital mortality when suffering from perioperative delirium (OR, 1.31, 95% CI: 1.28–1.33, *p* < 0.001), stroke (OR, 1.20, 95% CI: 1.17–1.23, *p* < 0.001), AMI (OR, 1.22, 95% CI: 1.19–1.25, *p* < 0.001) and AKI (OR, 1.15, 95% CI: 1.14–1.16, *p* < 0.001).

In addition, we found that neither age (OR: 1.02; 95% CI: 1.02–1.02, *p* < 0.001) nor chronic renal disease (OR: 0.94; 95% CI: 0.93–0.95, *p* < 0.001) or diabetes with (OR: 0.85; 95% CI: 0.84–0.86, *p* < 0.001) or without complications (OR: 0.93; 95% CI: 0.93–0.95, *p* < 0.001) is associated with a considerably risk of in-hospital mortality in any perioperative organ injury (Additional File 4).

## Discussion

In this retrospective cohort study, we investigated whether COPD patients with perioperative organ injury show a higher in-hospital mortality, HLOS and VT compared to patients without COPD. Perioperative organ injury was defined as the presence of perioperative delirium, stroke, AMI, ARDS, ALI, or AKI. We included adult hospitalised surgical patients with perioperative organ injuries in Germany between 2015 and 2019.

In 1.6 million analysed surgical cases with perioperative organ injury, we observed a higher in-hospital mortality, HLOS and VT in patients suffering from COPD compared to patients not suffering from COPD. Perioperative AKI was the most common perioperative organ injury, and perioperative ALI demonstrated the highest in-hospital mortality in patients suffering from COPD. To ascertain that the results observed are related to COPD, we performed different regression model analyses. Regression model analysis showed only a slightly higher risk of in-hospital mortality for COPD, when any perioperative organ injury is present. A more detailed analysis of different types of organ injuries revealed that COPD had also an adverse impact on patients suffering from delirium: in these patients, COPD was associated with the highest odds for in-hospital mortality, the longest HLOS as well as the longest VT compared to other perioperative organ injury.

Like our findings, a recent analysis of patients after elective surgery showed that COPD patients were more frequently of male gender, more likely suffering from pre-existing comorbidities and associated with an increased risk of death [25]. Fields et al. confirmed an increased mortality and demonstrated a prolonged duration of stay in COPD patients undergoing abdominal surgery [26]. The results of our study append the impact of COPD on surgical patients with perioperative organ injury to the existing body of evidence: COPD is not only associated with a higher in-hospital mortality and a longer HLOS, but also with a prolonged VT. Considering different types of perioperative organ injuries studied, we demonstrated that AKI was the most frequently observed perioperative organ injury in patients with and without COPD. While these results are supported by recent findings, Zhang et al. demonstrated that renal injury exists in patients with acute exacerbation of COPD, deteriorating with hypoxia [27]. Hypoxia may occur in COPD patients perioperative, resulting in aggravation of pre-existing renal impairment. This may explain the relatively high number of perioperative AKI in patients with COPD compared to patients without COPD. In addition, we observed perioperative delirium as the second most common perioperative organ injury in our retrospective analysis, fortunately with the lowest in-hospital mortality. An association between delirium and COPD is also already known, as Spiropoulou et al. demonstrated in cardiac surgery patients [28]. Additionally, cognitive impairment is present in COPD patients [29] resulting in an increased risk of hospitalisation when co-existing [30] and prolonged HLOS during COPD exacerbation [31]. Moreover, we detected the highest in-hospital mortality for ALI in patients with and without COPD. A meta-analysis reported that liver dysfunction in cardiac surgery is associated with higher short- and long-term mortality in cardiac surgery compared to patients with no liver dysfunction [32]. High mortality in acute liver dysfunction, as noticed in our retrospective analysis, is certainly due to limited perioperative treatment options.

However, our retrospective analysis also revealed unexpected results. First, we observed only a slightly higher prevalence and a lower in-hospital mortality for perioperative ARDS in COPD patients compared to patients without COPD. A possible explanation would be an inadequate coding, as a deterioration of the lung function was mostly not considered as perioperative ARDS, but as a worsening of the underlying COPD. However, other authors also reported unexpected results in COPD-patients. Liu et al. reported lower PEEP levels titrated by electrical impedance tomography in ARDS patients with compared to without COPD [33] and Azoulay et al. demonstrated that, among others, COPD was associated with a lower probability with invasive ventilation on admission day [34]. Moreover, Gadre et al. showed a lower ICU- and hospital mortality for ventilated patients suffering from ARDS based on an acute exacerbation of severe COPD compared with other causes of ARDS [35]. In addition, other pre-existing lung diseases like interstitial lung disease (ILD) might be higher in patients without COPD influencing in-hospital mortality. Second, the prevalence of AMI did not differ significantly between patients with and without COPD, although an association between COPD and coronary artery disease has already been demonstrated [36]. In addition, perioperative stroke and ALI were diagnosed more often in patients without COPD within the perioperative organ injuries studied. A possible explanation could be that COPD patients are treated with several medications due to frequent comorbidities, e.g. of cardiovascular nature. The medication could have preventive effect on the development of some complications. However, it could also be a purely coincidental observation. Third, our regression model analysis showed that COPD is associated with a slightly higher risk for in-hospital mortality. We found several comorbidities that had a higher impact on adverse outcome compared to COPD. We could demonstrate that SIRS/sepsis and moderate to severe liver disease were associated with considerably higher odds for in-hospital mortality. Due to the nature of our retrospective study design, we cannot rule out confounding between COPD and these other comorbidities. For example, a recently published meta-analysis revealed that COPD is associated with a higher risk for sepsis, septic shock, pneumonia, and surgical site infections within 30 days after hip arthroplasties [19]. Taking these two factors into consideration, perioperative infectious diseases may play an important role for patients with perioperative organ injury suffering from COPD. In addition, the occurrence of specific comorbidities associated with COPD, e.g., cardiovascular diseases, ischemic heart disease, arrhythmias, peripheral vascular disease, hypertension, and lung cancer, is already known and well-studied [14]. Any of these comorbidities may impact the outcome of COPD patients with perioperative organ injury.

Nevertheless, our results show that the presence of COPD in perioperative organ injury impacts the outcome regarding to in-hospital mortality, HLOS and VT. Therefore, clinicians should be alert and aware of the increased risk of this patient population. Knowing the more frequent prevalence and higher in-hospital mortality of different types of perioperative organ injuries in COPD patients, early aggressive therapy should be provided. Our results show that particular attention should be paid to perioperative delirium, the second most common perioperative organ failure in patients with and without COPD. However, perioperative delirium is associated with 5 days longer HLOS and 1.8 times (91 h) longer VT in patients with COPD compared to patients without COPD. Although in-hospital mortality was the lowest for perioperative delirium, we observed the second longest median HLOS in COPD patients. Our regression analysis demonstrated that in perioperative delirium, COPD is associated with the highest odds for in-hospital mortality, the longest HLOS and the most prolonged VT in perioperative organ injuries studied. This implicates that perioperative delirium has a major impact on recovery and should not be underestimated.

Some strength and weaknesses of this study must be highlighted. First, analysis of 1.6 million surgical cases with perioperative organ injury must be considered a strength. Second, we only analysed data before the pandemic so that no influence by a COVID-19 lung affection was present. Third, since in Germany reimbursement is based on mandatory coding of diagnosis, comorbidities and procedures, our results must be considered as reliable. Nevertheless, we cannot exclude inadequate coding entirely. In addition, there is a partial overlapping between the ICD codes used for calculating the CCI and the ICD codes selected for the definition of perioperative organ injury. Another important limitation is that only cases with perioperative organ injury were considered. Calculating the prevalence of each perioperative organ injury in entire surgical cases and the impact of COPD associated with each type of perioperative organ injury may lead to deviating results. In our study, patients were not categorised to different COPD severity grades. An analysis based on COPD severity grades including lung function parameter as well as the adequacy of perioperative COPD treatment could have provided interesting additional information. Furthermore, information about smoking status and body mass index were also not considered in this study. In addition to being a strength, large data analysis can also be seen as a weakness, as significances will result more easily. Another weakness may be that we investigated only data from one European country. Different perioperative hospitalisation periods in various countries may have an impact on in-hospital mortality. In addition, we only examined mortality for hospitalised patients. Mortality after discharge and outpatient care were not considered. And finally, we performed analysis for various surgical procedures. Dividing surgical procedures into emergency and elective surgery as well as low risk and high-risk surgeries would lead to interesting results certainly influencing in-hospital mortality.

Further investigations, such as prospective cohort studies, are needed to elicit the association of COPD and perioperative organ injury within emergency and elective surgery as well as within high or low risk surgery. Moreover, other perioperative organ injuries, e.g. pneumonia, should be emphasised in future studies. In addition, further research should highlight long-term mortality. Also, various countries with different hospitalisation periods should be illuminated to confirm our findings. Based on these results, individual perioperative risk stratification for COPD patients suffering from different types of perioperative organ injury can be performed.

## Conclusions

In summary, this retrospective analysis demonstrated that COPD is associated with higher in-hospital mortality, longer HLOS and longer VT in hospitalised surgical patients suffering from perioperative organ injury. Within the perioperative organ injuries analysed, AKI was the most common perioperative organ injury whereas ALI was associated with the highest mortality. Both perioperative organ injuries demonstrated a longer HLOS and VT in COPD patients compared to patients without COPD. Especially COPD patients may benefit from effective measures to prevent perioperative organ injuries in the future.

### Electronic supplementary material

Below is the link to the electronic supplementary material.

Supplementary Material 1

Supplementary Material 2

Supplementary Material 3

Supplementary Material 4

Supplementary Material 5

Supplementary Material 6

Supplementary Material 7

Supplementary Material 8

Supplementary Material 9

Supplementary Material 10

Supplementary Material 11

Supplementary Material 12

Supplementary Material 13

Supplementary Material 14

Supplementary Material 15

Supplementary Material 16

Supplementary Material 17

Supplementary Material 18

Supplementary Material 19

Supplementary Material 20

Supplementary Material 21

Supplementary Material 22

Supplementary Material 23

Supplementary Material 24

## Data Availability

We designed an analysis protocol as a Stata do-file and the analysis of the Stata do-file was performed on the actual data by the Federal Statistical Office. The actual data are not publicly available. Results were returned to the authors after a detailed review and curation of the data to avoid a possible de-anonymization of individuals.

## Abbreviations

COPD: Chronic Obstructive Pulmonary Disease
POI: Perioperative Organ Injury
AMI: Acute Myocardial Infarction
ARDS: Acute Respiratory Distress Syndrome
ALI: Acute Liver Injury
AKI: Acute Kidney Injury
HLOS: Hospital Length of Stay
VT: perioperative Ventilation Time
G-DRG: German Diagnosis-Related Groups
OPS: Operationen- und Prozedurenschlüssel
ICPM: International Classification of Procedures in Medicine
WHO: World Health Organization
CCI: Charlson Comorbidity Index
IQR: Interquartile ranges
AUROC: Area under the receiver operating curve
RMSE: Root mean square error
