# Supplementary material for: Chronic obstructive pulmonary disease affects outcome in surgical patients with perioperative organ injury: a retrospective cohort study in Germany
Source: Respir Res. 2024 Jun 20;25:251. doi: 10.1186/s12931-024-02882-3 (PMC11191349; doi:10.1186/s12931-024-02882-3)
Supplement: Supplementary file 1 — Supplementary Material 1 [file 12931_2024_2882_MOESM1_ESM.docx]

**Additional File 1:** Taxonomy of the *Operationen- und Prozedurenschlüssel* (OPS), the German version of the *International Classification of Procedures in Medicine* (ICPM), showing details of the subsections from “Chapter 5 – Operations”.

| Chapter 1 | Diagnostic Measures | |
| --- | --- | --- |
| Chapter 3 | Diagnostic Imaging | |
| Chapter 5 | Operations | |
|  | 5-01 to 5-05 | Operations on the Nervous System |
|  | 5-06 to 5-07 | Operations on Endocrine System |
|  | 5-08 to 5-16 | Operations on the Eyes |
|  | 5-18 to 5-20 | Operations on the Ears |
|  | 5-21 to 5-22 | Operations on Nose, Mouth and Pharynx |
|  | 5-23 to 5-28 | Operations in the Oral cavity and Face |
|  | 5-29 to 5-31 | Operations on Pharynx, Larynx and Trachea |
|  | 5-32 to 5-34 | Operations on the Lungs and Bronchi |
|  | 5-35 to 5-37 | Operations on the Heart |
|  | 5-38 to 5-39 | Operations on Blood Vessels |
|  | 5-40 to 5-41 | Operations on the Hemopoietic and Lymphatic Systems |
|  | 5-42 to 5-54 | Operations on the Digestive System |
|  | 5-55 to 5-59 | Operations on the Urinary Tract |
|  | 5-60 to 5-64 | Operations on Male Genital Organs |
|  | 5-65 to 5-71 | Operations on Female Genital Organs |
|  | 5-72 to 5-75 | Obstetric Operations |
|  | 5-76 to 5-77 | Maxillofacial Operations |
|  | 5-78 to 5-86 | Operations on the Musculoskeletal System |
|  | 5-87 to 5-88 | Operations on the Breasts |
|  | 5-89 to 5-92 | Operations on Skin and Subcutaneous Tissue |
|  | 5-93 to 5-99 | Additional Information on Operations |
| Chapter 6 | Medications | |
| Chapter 8 | Non-Surgical Therapeutic Measures | |
| Chapter 9 | Complimentary Measures | |
