# Supplementary material for: Chronic obstructive pulmonary disease affects outcome in surgical patients with perioperative organ injury: a retrospective cohort study in Germany
Source: Respir Res. 2024 Jun 20;25:251. doi: 10.1186/s12931-024-02882-3 (PMC11191349; doi:10.1186/s12931-024-02882-3)
Supplement: Supplementary file 2 — Supplementary Material 2 [file 12931_2024_2882_MOESM2_ESM.docx]

Additional File 2: Details of variable transcoding for diagnoses using ICD-10-GM codes.

| **Perioperative Organ Injuries** | |
| --- | --- |
| Delirium | F05 |
| Stroke | I63, I64 |
| Acute Myocardial Infarction | I21, I22 |
| Adult Respiratory Distress Syndrome | J80 |
| Acute Liver Injury | K71, K720 |
| Acute Kidney Injury | N17 |
| **Confounders** | |
| Pulmonary Embolism | I26 |
| Sepsis/SIRS | A40, A41, R65, A392, A393, A394 |
| **Comorbidities** | |
| Acute Myocardial Infarction | I21, I22, I252 |
| Congestive Heart Failure | I43, I50, I099, I110, I130, I132, I255, I420, I425, I426, I427, I428, I429, P290 |
| Peripheral Vascular Disease | I70, I71, I731, I738, I739, I771, I790, I792, K551, K558, K559, Z958, Z959, |
| Cerebrovascular Disease | G45, G46, I60, I61, I62, I63, I64, I65, I66, I67, I68, I69, H340 |
| Dementia | F00, F01, F02, F03, G30, F051, G311 |
| Chronic Pulmonary Disease | J40, J41, J42, J43, J44, J45, J46, J47, J60, J61, J62, J63, J64, J65, J66, J67, I278, I279, J684, J701, J703 |
| Rheumatologic Disease | M05, M32, M33, M34, M06, M315, M351, M353, M360 |
| Peptic Ulcer Disease | K25, K26, K27, K28 |
| Mild Liver Disease | B18, K73, K74, K700, K701, K702, K703, K709, K713, K714, K715, K717, K760, K762, K763, K764, K768, K769, Z944 |
| Moderate/Severe Liver Disease | K704, K711, K721, K729, K765, K766, K767, I850, I859, I864, I982 |
| Diabetes without complications | E100, E101, E106, E108, E109, E110, E111, E116, E118, E119, E120, E121, E126, E128, E129,E130, E131, E136, E138, E139, E140, E141, E146, E148, E149 |
| Diabetes with chronic complications | E102, E103, E104, E105, E107, E112, E113 , E114, E115, E117, E122, E123, E124, E125, E127,E132, E133, E134, E135, E137, E142, E143, E144, E145, E147 |
| Hemiplegia or Paraplegia | G81, G82, G041, G114, G801, G802, G830, G831, G832, G833, G834, G839 |
| Renal Disease | N18, N19, N052, N053, N054, N055, N056, N057, N250, I120, I131, N032, N033, N034, N035, N036, N037, Z490, Z491, Z492, Z940, Z992 |
| Cancer | C00, C01, C02, C03, C04, C05, C06, C07, C08, C09, C10, C11, C12, C13, C14, C15, C16, C17, C18, C19, C20, C21, C22, C23, C24, C25, C26, C30, C31, C32, C33, C34, C37, C38, C39, C40, C41, C43, C45, C46, C47, C48, C49, C50, C51, C52, C53, C54, C55, C56, C57, C58, C60, C61, C62, C63, C64, C65, C66, C67, C68, C69, C70, C71, C72, C73, C74, C75, C76, C81, C82, C83, C84, C85, C88, C90, C91, C92, C93, C94, C95, C96, C97 |
| Metastatic Cancer | C77, C78, C79, C80 |
| AIDS/HIV | B20, B21, B22, B24 |

SIRS: Systemic Inflammatory Response Syndrome, AIDS: acquired immune deficiency syndrome, HIV: Human immunodeficiency virus.
