# Supplementary material for: Chronic obstructive pulmonary disease affects outcome in surgical patients with perioperative organ injury: a retrospective cohort study in Germany
Source: Respir Res. 2024 Jun 20;25:251. doi: 10.1186/s12931-024-02882-3 (PMC11191349; doi:10.1186/s12931-024-02882-3)
Supplement: Supplementary file 3 — Supplementary Material 3 [file 12931_2024_2882_MOESM3_ESM.docx]

Additional File 3: Operations on different organ systems of 1,642,377 hospitalised surgical patients suffering from perioperative organ injury with chronic obstructive pulmonary disease (COPD) and without COPD.

| Operations* | All patients  (N = 1,642,377) | Patients with COPD  (N = 177,070; 10.8%) | Patients without COPD  (N = 1,465,307; 89.2%) | P-value COPD vs no-COPD |
| --- | --- | --- | --- | --- |
| Nervous system- no (%) | 104,932 (6.4) | 7,120 (4.0) | 97,812 (6.7) | <0.001 |
| Endocrine system- no (%) | 7,262 (0.4) | 854 (0.5) | 6,408 (0.4) | 0.007 |
| Eyes- no (%) | 7,145 (0.4) | 485 (0.3) | 6,660 (0.5) | <0.001 |
| Ears- no (%) | 3,011 (0.2) | 226 (0.1) | 2,785 (0.2) | <0.001 |
| Nose, Mouth and Pharynx- no (%) | 8,118 (0.5) | 832 (0.5) | 7,286 (0.5) | 0.121 |
| Oral cavity and face- no (%) | 15,559 (1.0) | 1,591 (0.9) | 13,968 (1.0) | 0.025 |
| Pharynx, larynx, and trachea- no (%) | 144,966 (8.8) | 28,921 (16.3) | 116,045 (8.0) | <0.001 |
| Lungs and bronchi- no (%) | 73,543 (4.5) | 12,136 (6.9) | 61,407 (4.2) | <0.001 |
| Heart- no (%) | 268,707 (16.4) | 28,024 (15.8) | 240,683 (16.4) | <0.001 |
| Blood vessels- no (%) | 259,993 (15.8) | 30,220 (17.1) | 229,773 (15.7) | <0.001 |
| Hemopoietic and lymphatic- no (%) | 42,149 (2.6) | 3,925 (2.2) | 38,224 (2.6) | <0.001 |
| Digestive system- no (%) | 532,958 (32.5) | 58,568 (33.1) | 474,390 (32.4) | <0.001 |
| Urinary tract- no (%) | 153,665 (9.4) | 12,703 (7.2) | 140,962 (9.6) | <0.001 |
| Male genital organs- no (%) | 24,967 (1.5) | 1,977 (1.1) | 22,990 (1.6) | <0.001 |
| Female genital organs- no (%) | 16,483 (1.0) | 1,077 (0.6) | 15,406 (1.1) | <0.001 |
| Obstetric- no (%) | 2,392 (0.15) | XXX | XXX |  |
| Maxillofacial- no (%) | 6,032 (0.4) | 417 (0.2) | 5,615 (0.4) | <0.001 |
| Musculoskeletal system- no (%) | 415,768 (25.3) | 40,430 (22.8) | 375,338 (25.6) | <0.001 |
| Breasts- no (%) | 2,413 (0.2) | 173 (0.1) | 2,240 (0.2) | <0.001 |
| Skin and subcutaneous tissue- no (%) | 249,040 (15.2) | 28,002 (15.8) | 221,038 (15.1) | <0.001 |

no: number, IQR: interquartile range.

XXX: For reasons of data protection, this number was not published.

*non-exclusive categories due to multiple surgeries or multiple surgical sites during one surgery
