# Supplementary material for: Chronic obstructive pulmonary disease affects outcome in surgical patients with perioperative organ injury: a retrospective cohort study in Germany
Source: Respir Res. 2024 Jun 20;25:251. doi: 10.1186/s12931-024-02882-3 (PMC11191349; doi:10.1186/s12931-024-02882-3)
Supplement: Supplementary file 4 — Supplementary Material 4 [file 12931_2024_2882_MOESM4_ESM.docx]

Additional File 4. Risk-adjusted associations of **In-hospital mortality** from multivariable regression analysis models analysing the impact of COPD in 1,642,375 hospitalized surgical patients with any perioperative organ injury.

|  | Odds ratio (95% CI) | P- value |
| --- | --- | --- |
| COPD | 1.19 (1.18-1.21) | <0.001 |
| Age | 1.02 (1.02-1.02) | <0.001 |
| Female | 1.07 (1.06-1.08) | <0.001 |
| Emergency hospital admission | 1.23 (1.22-1.24) | <0.001 |
| *Charlson comorbidity score items* | | |
| Myocardial infarction | 1.06 (1.05-1.07) | <0.001 |
| Chronic heart failure | 1.56 (1.54-1.57) | <0.001 |
| Peripheral vascular disease | 1.40 (1.38-1.42) | <0.001 |
| Cerebrovascular disease | 1.17 (1.16-1.19) | <0.001 |
| Dementia | 0.89 (0.88-0.90) | <0.001 |
| Rheumatic disease | 0.96 (0.93-1.00) | 0.034 |
| Peptic ulcer disease | 1.59 (1.56-1.62) | <0.001 |
| Mild liver disease | 1.57 (1.54-1.60) | <0.001 |
| Moderate to severe liver disease | 3.80 (3.72-3.89) | <0.001 |
| Diabetes without complications | 0.93 (0.92-0.94) | <0.001 |
| Diabetes with complications | 0.85 (0.84-0.86) | <0.001 |
| Paraplegia or hemiplegia | 0.87 (0.86-0.89) | <0.001 |
| Renal disease | 0.94 (0.93-0.95) | <0.001 |
| Cancer | 1.41 (1.39-1.43) | <0.001 |
| Metastatic cancer | 2.69 (2.65-2.73) | <0.001 |
| AIDS | 1.84 (1.60-2.11) | <0.001 |
| Pulmonary embolism | 2.18 (2.12-2.24) | <0.001 |
| Sepsis/ SIRS | 4.52 (4.47-4.56) | <0.001 |
