# Supplementary material for: Chronic obstructive pulmonary disease affects outcome in surgical patients with perioperative organ injury: a retrospective cohort study in Germany
Source: Respir Res. 2024 Jun 20;25:251. doi: 10.1186/s12931-024-02882-3 (PMC11191349; doi:10.1186/s12931-024-02882-3)
Supplement: Supplementary file 5 — Supplementary Material 5 [file 12931_2024_2882_MOESM5_ESM.docx]

Additional File 5. Risk-Adjusted associations of **In-hospital mortality** from multivariable regression analysis models analysing the impact of COPD in 571,526 hospitalized surgical patients with perioperative delirium.

|  | Odds ratio (95% CI) | P- value |
| --- | --- | --- |
| COPD | 1.30 (1.27-1.34) | <0.001 |
| Age | 1.03 (1.03-1.03) | <0.001 |
| Female | 0.85 (0.83-0.86) | <0.001 |
| Emergency hospital admission | 1.31 (1.28-1.33) | <0.001 |
| *Charlson comorbidity score items* | | |
| Myocardial infarction | 1.03 (0.99-1.07) | 0.187 |
| Chronic heart failure | 1.54 (1.51-1.57) | <0.001 |
| Peripheral vascular disease | 1.36 (1.33-1.39) | <0.001 |
| Cerebrovascular disease | 0.99 (0.96-1.02) | 0.588 |
| Dementia | 0.97 (0.95-0.99) | 0.006 |
| Rheumatic disease | 1.10 (1.03-1.17) | 0.005 |
| Peptic ulcer disease | 1.65 (1.59-1.71) | <0.001 |
| Mild liver disease | 1.36 (1.30-1.41) | <0.001 |
| Moderate to severe liver disease | 2.80 (2.64-2.98) | <0.001 |
| Diabetes without complications | 0.95 (0.93-0.97) | <0.001 |
| Diabetes with complications | 1.01 (0.98-1.04) | 0.559 |
| Paraplegia or hemiplegia | 1.07 (1.04-1.11) | <0.001 |
| Renal disease | 1.18 (1.15-1.20) | <0.001 |
| Cancer | 1.70 (1.66-1.75) | <0.001 |
| Metastatic cancer | 3.51 (3.41-3.62) | <0.001 |
| AIDS | 2.05 (1.54-2.72) | <0.001 |
| Pulmonary embolism | 2.01 (1.91-2.13) | <0.001 |
| Sepsis/SIRS | 2.81 (2.75-2.87) | <0.001 |
| POI Stroke | 1.50 (1.43-1.58) | <0.001 |
| POI AMI | 1.18 (1.12-1.24) | <0.001 |
| POI ARDS | 1.84 (1.75-1.94) | <0.001 |
| POI ALI | 3.93 (3.75-4.12) | <0.001 |
| POI AKI | 2.43 (2.38-2.48) | <0.001 |

POI Stroke - Perioperative stroke; POI AMI - Perioperative acute myocardial infarction; POI ARDS - Perioperative acute respiratory distress syndrome; POI ALI - Perioperative acute liver injury; POI AKI - Perioperative acute kidney injury
