# Supplementary material for: Chronic obstructive pulmonary disease affects outcome in surgical patients with perioperative organ injury: a retrospective cohort study in Germany
Source: Respir Res. 2024 Jun 20;25:251. doi: 10.1186/s12931-024-02882-3 (PMC11191349; doi:10.1186/s12931-024-02882-3)
Supplement: Supplementary file 6 — Supplementary Material 6 [file 12931_2024_2882_MOESM6_ESM.docx]

Additional File 6. Risk-Adjusted associations of **In-hospital mortality** from multivariable regression analysis models analysing the impact of COPD in 209,910 hospitalized surgical patients with perioperative stroke.

|  | Odds ratio (95% CI) | P- value |
| --- | --- | --- |
| COPD | 1.15 (1.09-1.20) | <0.001 |
| Age | 1.02 (1.01-1.02) | <0.001 |
| Female | 1.18 (1.15-1.21) | <0.001 |
| Emergency hospital admission | 1.20 (1.17-1.23) | <0.001 |
| *Charlson comorbidity score items* | | |
| Myocardial infarction | 0.98 (0.92-1.05) | 0.656 |
| Chronic heart failure | 1.35 (1.30-1.39) | <0.001 |
| Peripheral vascular disease | 1.21 (1.17-1.26) | <0.001 |
| Dementia | 1.08 (1.03-1.13) | 0.003 |
| Rheumatic disease | 0.84 (0.75-0.94) | 0.002 |
| Peptic ulcer disease | 1.27 (1.19-1.36) | <0.001 |
| Mild liver disease | 1.16 (1.07-1.25) | <0.001 |
| Moderate to severe liver disease | 1.94 (1.69-2.22) | <0.001 |
| Diabetes without complications | 0.94 (0.91-0.97) | <0.001 |
| Diabetes with complications | 0.93 (0.88-0.98) | 0.005 |
| Paraplegia or hemiplegia | 0.71 (0.69-0.73) | <0.001 |
| Renal disease | 1.12 (1.08-1.16) | <0.001 |
| Cancer | 1.50 (1.42-1.59) | <0.001 |
| Metastatic cancer | 2.69 (2.53-2.85) | <0.001 |
| AIDS | 1.90 (1.17-3.07) | 0.010 |
| Pulmonary embolism | 1.97 (1.82-2.13) | <0.001 |
| Sepsis/SIRS | 2.66 (2.57-2.76) | <0.001 |
| POI Delirium | 0.70 (0.67-0,73) | <0.001 |
| POI AMI | 1.52 (1.40-1.65) | <0.001 |
| POI ARDS | 2.04 (1.85-2.24) | <0.001 |
| POI ALI | 3.44 (3.12-3.79) | <0.001 |
| POI AKI | 2.86 (2.76-2.96) | <0.001 |

Cerebrovascular Disease was omitted because of collinearity.

POI Delirium - Perioperative delirium; POI AMI - Perioperative acute myocardial infarction; POI ARDS - Perioperative acute respiratory distress syndrome; POI ALI - Perioperative acute liver injury; POI AKI - Perioperative acute kidney injury
