# Supplementary material for: Chronic obstructive pulmonary disease affects outcome in surgical patients with perioperative organ injury: a retrospective cohort study in Germany
Source: Respir Res. 2024 Jun 20;25:251. doi: 10.1186/s12931-024-02882-3 (PMC11191349; doi:10.1186/s12931-024-02882-3)
Supplement: Supplementary file 7 — Supplementary Material 7 [file 12931_2024_2882_MOESM7_ESM.docx]

Additional File 7. Risk-Adjusted associations of **In-hospital mortality** from multivariable regression analysis models analysing the impact of COPD in 212,994 hospitalized surgical patients with perioperative acute myocardial infarction.

|  | Odds ratio (95% CI) | P- value |
| --- | --- | --- |
| COPD | 1.10 (1.06-1.14) | <0.001 |
| Age | 1.03 (1.03-1.03) | <0.001 |
| Female | 1.09 (1.06-1.12) | <0.001 |
| Emergency hospital admission | 1.22 (1.19-1.25) | <0.001 |
| *Charlson comorbidity score items* | | |
| Chronic heart failure | 1.08 (1.06-1.11) | <0.001 |
| Peripheral vascular disease | 1.55 (1.50-1.59) | <0.001 |
| Cerebrovascular disease | 1.04 (1.00-1.09) | 0.076 |
| Dementia | 1.58 (1.50-1.67) | <0.001 |
| Rheumatic disease | 0.89 (0.81-0.99) | 0.029 |
| Peptic ulcer disease | 1.19 (1.12-1.26) | <0.001 |
| Mild liver disease | 1.19 (1.10-1.28) | <0.001 |
| Moderate to severe liver disease | 1.97 (1.75-2.21) | <0.001 |
| Diabetes without complications | 0.89 (0.86-0.92) | <0.001 |
| Diabetes with complications | 0.92 (0.88-0.96) | <0.001 |
| Paraplegia or hemiplegia | 0.94 (0.88-0.99) | 0.031 |
| Renal disease | 0.93 (0.90-0.96) | <0.001 |
| Cancer | 1.42 (1.35-1.50) | <0.001 |
| Metastatic cancer | 2.16 (2.02-2.32) | <0.001 |
| AIDS | 1.31 (0.71-2.41) | 0.392 |
| Pulmonary embolism | 1.62 (1.48-1.76) | <0.001 |
| Sepsis/SIRS | 2.59 (2.50-2.67) | <0.001 |
| POI Delirium | 0.63 (0.61-0.66) | <0.001 |
| POI Stroke | 1.74 (1.62-1.86) | <0.001 |
| POI ARDS | 2.61 (2.40-2.85) | <0.001 |
| POI ALI | 6.46 (6.01-6.95) | <0.001 |
| POI AKI | 3.02 (2.94-3.11) | <0.001 |

Acute myocardial Infarction was omitted because of collinearity.

POI Delirium - Perioperative delirium; POI Stroke - Perioperative stroke; POI ARDS - Perioperative acute respiratory distress syndrome; POI ALI - Perioperative acute liver injury; POI AKI - Perioperative acute kidney injury
