# Supplementary material for: Chronic obstructive pulmonary disease affects outcome in surgical patients with perioperative organ injury: a retrospective cohort study in Germany
Source: Respir Res. 2024 Jun 20;25:251. doi: 10.1186/s12931-024-02882-3 (PMC11191349; doi:10.1186/s12931-024-02882-3)
Supplement: Supplementary file 8 — Supplementary Material 8 [file 12931_2024_2882_MOESM8_ESM.docx]

Additional File 8. Risk-Adjusted associations of **In-hospital mortality** from multivariable regression analysis models analysing the impact of COPD in 39,723 hospitalized surgical patients with perioperative acute respiratory distress syndrome.

|  | Odds ratio (95% CI) | P- value |
| --- | --- | --- |
| COPD | 0.82 (0.77-0.88) | <0.001 |
| Age | 1.04 (1.04-1.04) | <0.001 |
| Female | 1.01 (0.96-1.05) | 0.930 |
| Emergency hospital admission | 1.01 (0.96-1.05) | 0.737 |
| *Charlson comorbidity score items* | | |
| Myocardial infarction | 1.09 (0.96-1.23) | 0.183 |
| Chronic heart failure | 1.08 (1.03-1.13) | 0.002 |
| Peripheral vascular disease | 1.34 (1.26-1.42) | <0.001 |
| Cerebrovascular disease | 1.47 (1.36-1.60) | <0.001 |
| Dementia | 1.40 (1.21-1.63) | <0.001 |
| Rheumatic disease | 1.14 (0.97-1.33) | 0.115 |
| Peptic ulcer disease | 1.16 (1.06-1.27) | 0.002 |
| Mild liver disease | 1.25 (1.16-1.36) | <0.001 |
| Moderate to severe liver disease | 2.95 (2.62-3.32) | <0.001 |
| Diabetes without complications | 0.83 (0.78-0.87) | <0.001 |
| Diabetes with complications | 0.80 (0.73-0.88) | <0.001 |
| Paraplegia or hemiplegia | 0.45 (0.41-0.49) | <0.001 |
| Renal disease | 1.10 (1.00-1.12) | 0.054 |
| Cancer | 1.89 (1.76-2.02) | <0.001 |
| Metastatic cancer | 2.59 (2.36-2.84) | <0.001 |
| AIDS | 1.73 (1.27-2.36) | 0.001 |
| Pulmonary embolism | 1.23 (1.12-1.36) | <0.001 |
| Sepsis/SIRS | 1.29 (1.23-1.36) | <0.001 |
| POI Delirium | 0.35 (0.33-0.37) | <0.001 |
| POI Stroke | 1.01( 0.89-1.13) | 0.926 |
| POI AMI | 1.03 (0.89-1.19) | 0.675 |
| POI ALI | 4.01 (3.73-4.31) | <0.001 |
| POI AKI | 2.01 (1.92-2.12) | <0.001 |

POI Delirium - Perioperative delirium; POI Stroke - Perioperative stroke; POI AMI - Perioperative acute myocardial infarction; POI ALI - Perioperative acute liver injury; POI AKI - Perioperative acute kidney injury
