# Supplementary material for: Chronic obstructive pulmonary disease affects outcome in surgical patients with perioperative organ injury: a retrospective cohort study in Germany
Source: Respir Res. 2024 Jun 20;25:251. doi: 10.1186/s12931-024-02882-3 (PMC11191349; doi:10.1186/s12931-024-02882-3)
Supplement: Supplementary file 9 — Supplementary Material 9 [file 12931_2024_2882_MOESM9_ESM.docx]

Additional File 9. Risk-Adjusted associations of **In-hospital mortality** from multivariable regression analysis models analysing the impact of COPD in 87,485 hospitalized surgical patients with perioperative acute liver injury.

|  | Odds ratio (95% CI) | P- value |
| --- | --- | --- |
| COPD | 1.16 (1.10-1.23) | <0.001 |
| Age | 1.02 (1.02-1.02) | <0.001 |
| Female | 0.96 (0.93-0.99) | 0.021 |
| Emergency hospital admission | 1.03 (1.03-1.07) | 0.049 |
| *Charlson comorbidity score items* | | |
| Myocardial infarction | 1.10 (1.01-1.20) | 0.038 |
| Chronic heart failure | 1.34 (1.29-1.40) | <0.001 |
| Peripheral vascular disease | 1.63 (1.57-1.71) | <0.001 |
| Cerebrovascular disease | 1.34 (1.24-1.44) | <0.001 |
| Dementia | 0.93 (0.85-1.02) | 0.109 |
| Rheumatic disease | 0.81 (0.72-0.93) | 0.001 |
| Peptic ulcer disease | 1.38 (1.29-1.47) | <0.001 |
| Mild liver disease | 1.16 (1.10-1.21) | <0.001 |
| Moderate to severe liver disease | 1.54 (1.46-1.62) | <0.001 |
| Diabetes without complications | 0.90 (0.87-0.94) | <0.001 |
| Diabetes with complications | 0.81 (0.76-0.87) | <0.001 |
| Paraplegia or hemiplegia | 0.67 (0.62-0.73) | <0.001 |
| Renal Disease | 1.16 (1.11-1.20) | <0.001 |
| Cancer | 1.24 (1.18-1.30) | <0.001 |
| Metastatic cancer | 2.03 (1.94-2.13) | <0.001 |
| AIDS | 1.12 (0.80-1.56) | 0.518 |
| Pulmonary embolism | 1.52 (1.38-1.68) | <0.001 |
| Sepsis/SIRS | 2.79 (2.69-2.89) | <0.001 |
| POI Delirium | 0.53 (0.50-0.55) | <0.001 |
| POI Stroke | 1.14 (1.01-1.29) | 0.036 |
| POI AMI | 1.61 (1.44-1.81) | <0.001 |
| POI ARDS | 2.18 (2.03-2.34) | <0.001 |
| POI AKI | 6.32 (6.10-6.55) | <0.001 |

POI Delirium - Perioperative delirium; POI Stroke - Perioperative stroke; POI AMI - Perioperative acute myocardial infarction; POI ARDS - Perioperative acute respiratory distress syndrome; POI AKI - Perioperative acute kidney injury
