# Supplementary material for: Chronic obstructive pulmonary disease affects outcome in surgical patients with perioperative organ injury: a retrospective cohort study in Germany
Source: Respir Res. 2024 Jun 20;25:251. doi: 10.1186/s12931-024-02882-3 (PMC11191349; doi:10.1186/s12931-024-02882-3)
Supplement: Supplementary file 10 — Supplementary Material 10 [file 12931_2024_2882_MOESM10_ESM.docx]

Additional File 10. Risk-Adjusted associations of **In-hospital mortality** from multivariable regression analysis models analysing the impact of COPD in 840,424 hospitalized surgical patients with perioperative acute kidney injury.

|  | Odds ratio (95% CI) | P- value |
| --- | --- | --- |
| COPD | 1.22 (1.20-1.24) | <0.001 |
| Age | 1.03 (1.03-1.03) | <0.001 |
| Female | 1.06 (1.05-1.07) | <0.001 |
| Emergency hospital admission | 1.15 (1.14-1.16) | <0.001 |
| *Charlson comorbidity score items* | | |
| Myocardial infarction | 0.99 (0.97-1.02) | 0.457 |
| Chronic heart failure | 1.44 (1.42-1.46) | <0.001 |
| Peripheral vascular disease | 1.40 (1.38-1.42) | <0.001 |
| Cerebrovascular disease | 1.20 (1.17-1.22) | <0.001 |
| Dementia | 1.29 (1.26-1.31) | <0.001 |
| Rheumatic disease | 0.98 (0.94-1.02) | 0.343 |
| Peptic Ulcer disease | 1.41 (1.39-1.45) | <0.001 |
| Mild liver disease | 1.39 (1.36-1.43) | <0.001 |
| Moderate to severe liver disease | 3.03 (2.95-3.11) | <0.001 |
| Diabetes without complications | 0.91 (0.90-0.93) | <0.001 |
| Diabetes with complications | 0.79 (0.77-0.80) | <0.001 |
| Paraplegia or hemiplegia | 0.90 (0.87-0.92) | <0.001 |
| Renal disease | 0.73 (0.72-0.74) | <0.001 |
| Cancer | 1.23 (1.21-1.25) | <0.001 |
| Metastatic cancer | 2.35 (2.31-2.40) | <0.001 |
| AIDS | 1.69 (1.41-2.01) | <0.001 |
| Pulmonary embolism | 1.93 (1.86-2.00) | <0.001 |
| Sepsis/SIRS | 3.33 (3.29-3.37) | <0.001 |
| POI Delirium | 0.80 (0.79-0.82) | <0.001 |
| POI Stroke | 1.70 (1.64-1.76) | <0.001 |
| POI AMI | 1.70 (1.64-1.75) | <0.001 |
| POI ARDS | 2.79 (2.71-2.88) | <0.001 |
| POI ALI | 6.68 (6.52-6.84) | <0.001 |

POI Delirium - Perioperative delirium; POI Stroke - Perioperative stroke; POI AMI - Perioperative acute myocardial infarction; POI ARDS - Perioperative acute respiratory distress syndrome; POI ALI - Perioperative acute liver injury
