# Supplementary material for: Chronic obstructive pulmonary disease affects outcome in surgical patients with perioperative organ injury: a retrospective cohort study in Germany
Source: Respir Res. 2024 Jun 20;25:251. doi: 10.1186/s12931-024-02882-3 (PMC11191349; doi:10.1186/s12931-024-02882-3)
Supplement: Supplementary file 11 — Supplementary Material 11 [file 12931_2024_2882_MOESM11_ESM.docx]

Additional File 11. Risk-Adjusted associations of **Hospital length of stay** from multivariable regression analysis models analysing the impact of COPD in 1,642,375 hospitalized surgical patients with any perioperative organ injury.

|  | Coefficient (95% CI) | P- value |
| --- | --- | --- |
| COPD | 2.62 (2.51-2.73) | <0.001 |
| Age | -0.16 (-0.17- -0.16) | <0.001 |
| Female | 0.83 (0.77-0.90) | <0.001 |
| Emergency hospital admission | -1.01 (-1.07- -0.94) | <0.001 |
| *Charlson comorbidity score items* | | |
| Myocardial infarction | -2.68 (-2.76- -2.60) | <0.001 |
| Chronic heart failure | 4.37 (4.30-4.44) | <0.001 |
| Peripheral vascular disease | 3.13 (3.04-3.22) | <0.001 |
| Cerebrovascular disease | -0.30 (-0.40- -0.19) | <0.001 |
| Dementia | -0.85 (-0.93- -0.77) | <0.001 |
| Rheumatic disease | 3.05 (2.79-3.32) | <0.001 |
| Peptic ulcer disease | 4.62 (4.43-4.81) | <0.001 |
| Mild liver disease | 1.49 (1.31-1.67) | <0.001 |
| Moderate to severe liver disease | 2.00 (1.74-2.25) | <0.001 |
| Diabetes without complications | 1.03 (0.95-1.11) | <0.001 |
| Diabetes with complications | 1.89 (1.78-2.01) | <0.001 |
| Paraplegia or hemiplegia | 7.53 (7.38-7.69) | <0.001 |
| Renal disease | 1.44 (1.37-1.52) | <0.001 |
| Cancer | 2.85 (2.74-2.95) | <0.001 |
| Metastatic cancer | 3.06 (2.94-3.19) | <0.001 |
| AIDS | 10.85 (8.95-12.75) | <0.001 |
| Pulmonary embolism | 7.10 (6.77-7.43) | <0.001 |
| Sepsis/SIRS | 13.06 (12.94-13.17) | <0.001 |
