# Supplementary material for: Chronic obstructive pulmonary disease affects outcome in surgical patients with perioperative organ injury: a retrospective cohort study in Germany
Source: Respir Res. 2024 Jun 20;25:251. doi: 10.1186/s12931-024-02882-3 (PMC11191349; doi:10.1186/s12931-024-02882-3)
Supplement: Supplementary file 12 — Supplementary Material 12 [file 12931_2024_2882_MOESM12_ESM.docx]

Additional File 12. Risk-Adjusted associations of **Hospital length of stay** from multivariable regression analysis models analysing the impact of COPD in 571,526 hospitalized surgical patients with perioperative delirium.

|  | Coefficient (95% CI) | P- value |
| --- | --- | --- |
| COPD | 2.80 (2.60-2.99) | <0.001 |
| Age | -0.26 (-0.27- -0.26) | <0.001 |
| Female | 1.16 (1.05-1.28) | <0.001 |
| Emergency hospital admission | -0.04 (-0.16-0.07) | 0.469 |
| *Charlson comorbidity score items* | | |
| Myocardial infarction | -0.67 (-0.91- -0.42) | <0.001 |
| Chronic heart failure | 3.21 (3.09-3.34) | <0.001 |
| Peripheral vascular disease | 2.76 (2.60-2.92) | <0.001 |
| Cerebrovascular disease | 0.18 (-0.01-0.37) | 0.060 |
| Dementia | -2.88 (-2.98- -2.77) | <0.001 |
| Rheumatic disease | 3.23 (2.77-3.69) | <0.001 |
| Peptic ulcer disease | 5.85 (5.47-6.23) | <0.001 |
| Mild liver disease | 2.72 (2.33-3.12) | <0.001 |
| Moderate to severe liver disease | 4.14 (3.36-4.92) | <0.001 |
| Diabetes without complications | 0.89 (0.75-1.03) | <0.001 |
| Diabetes with complications | 1.81 (1.60-2.03) | <0.001 |
| Paraplegia or hemiplegia | 7.32 (7.01-7.63) | <0.001 |
| Renal disease | 1.29 (1.16-1.42) | <0.001 |
| Cancer | 3.56 (3.36-3.76) | <0.001 |
| Metastatic cancer | 4.09 (3.85-4.33) | <0.001 |
| AIDS | 9.26 (5.39-13.14) | <0.001 |
| Pulmonary embolism | 7.55 (6.92-8.17) | <0.001 |
| Sepsis/SIRS | 14.54 (14.31-14.77) | <0.001 |
| POI Stroke | 3.85 (3.45-4.26) | <0.001 |
| POI AMI | 0.02 (-0.32-0.37) | 0.900 |
| POI ARDS | 8.23 (7.50-8.96) | <0.001 |
| POI ALI | 2.60 (1.87-3.32) | <0.001 |
| POI AKI | 5.43 (5.27-5.60) | <0.001 |

POI Stroke - Perioperative stroke; POI AMI - Perioperative acute myocardial infarction; POI ARDS - Perioperative acute respiratory distress syndrome; POI ALI - Perioperative acute liver injury; POI AKI - Perioperative acute kidney injury
