# Supplementary material for: Chronic obstructive pulmonary disease affects outcome in surgical patients with perioperative organ injury: a retrospective cohort study in Germany
Source: Respir Res. 2024 Jun 20;25:251. doi: 10.1186/s12931-024-02882-3 (PMC11191349; doi:10.1186/s12931-024-02882-3)
Supplement: Supplementary file 13 — Supplementary Material 13 [file 12931_2024_2882_MOESM13_ESM.docx]

Additional File 13. Risk-Adjusted associations of **Hospital length of stay** from multivariable regression analysis models analysing the impact of COPD in 209,910 hospitalized surgical patients with perioperative stroke.

|  | Coefficient (95% CI) | P- value |
| --- | --- | --- |
| COPD | 2.57 (2.14-3.00) | <0.001 |
| Age | -0.12 (-0.13- -0.12) | <0.001 |
| Female | 0.71 (0.51- 0.92) | <0.001 |
| Emergency hospital admission | -5.42 (-5.64- -5.20) | <0.001 |
| *Charlson comorbidity score items* | | |
| Myocardial infarction | -1.05 (-1.57- -0.54) | <0.001 |
| Chronic heart failure | 3.20 (2.93-3.48) | <0.001 |
| Peripheral vascular disease | 1.37 (1.08-1.67) | <0.001 |
| Dementia | -0.76 (-1.05- -0.46) | <0.001 |
| Rheumatic disease | 1.95 (1.08-2.83) | <0.001 |
| Peptic ulcer disease | 6.24 (5.55-6.94) | <0.001 |
| Mild liver disease | 3.65 (2.86-4.45) | <0.001 |
| Moderate to severe liver disease | 4.61 (2.77-6.45) | <0.001 |
| Diabetes without complications | 1.63 (1.38-1.88) | <0.001 |
| Diabetes with complications | 2.35 (1.93-2.76) | <0.001 |
| Paraplegia or hemiplegia | 7.44 (7.24-7.63) | <0.001 |
| Renal disease | 1.24 (0.96-1.51) | <0.001 |
| Cancer | 2.91 (2.44-3.38) | <0.001 |
| Metastatic cancer | 2.28 (1.77-2.80) | <0.001 |
| AIDS | 8.73 (3.09-14.37) | 0.002 |
| Pulmonary embolism | 5.33 (4.44-6.21) | <0.001 |
| Sepsis/SIRS | 15.21 (14.69-15.73) | <0.001 |
| POI Delirium | 9.05 (8.67-9.43) | <0.001 |
| POI AMI | 1.36 (0.64-2.08) | <0.001 |
| POI ARDS | 9.30 (7.75-10.85) | <0.001 |
| POI ALI | -1.45 (-2.94-0.03) | 0.055 |
| POI AKI | 2.81 (2.41-3.21) | <0.001 |

Cerebrovascular disease was omitted because of collinearity.

POI Delirium - Perioperative delirium; POI AMI - Perioperative acute myocardial infarction; POI ARDS - Perioperative acute respiratory distress syndrome; POI ALI - Perioperative acute liver injury; POI AKI - Perioperative acute kidney injury
