# Supplementary material for: Chronic obstructive pulmonary disease affects outcome in surgical patients with perioperative organ injury: a retrospective cohort study in Germany
Source: Respir Res. 2024 Jun 20;25:251. doi: 10.1186/s12931-024-02882-3 (PMC11191349; doi:10.1186/s12931-024-02882-3)
Supplement: Supplementary file 14 — Supplementary Material 14 [file 12931_2024_2882_MOESM14_ESM.docx]

Additional File 14. Risk-Adjusted associations of **Hospital length of stay** from multivariable regression analysis models analysing the impact of COPD in 212,994 hospitalized surgical patients with perioperative acute myocardial infarction.

|  | Coefficient (95% CI) | P- value |
| --- | --- | --- |
| COPD | 2.31 (2.05-2.57) | <0.001 |
| Age | -0.08 (-0.08- -0.07) | <0.001 |
| Female | 1.52 (1.36-1.68) | <0.001 |
| Emergency hospital admission | 0.97 (0.82-1.12) | <0.001 |
| *Charlson comorbidity score items* | | |
| Chronic heart failure | 2.36 (2.21-2.50) | <0.001 |
| Peripheral vascular disease | 2.93 (2.73-3.13) | <0.001 |
| Cerebrovascular disease | 0.02 (-0.27-10.31) | 0.886 |
| Dementia | -0.80 (-1.21- -0.47) | <0.001 |
| Rheumatic disease | 2.88 (2.24-3.51) | <0.001 |
| Peptic ulcer disease | 4.91 (4.39-5.42) | <0.001 |
| Mild liver disease | 2.68 (2.07-3.29) | <0.001 |
| Moderate to severe liver disease | 2.37 (1.06-3.67) | <0.001 |
| Diabetes without complications | 0.63 (0.46-0.79) | <0.001 |
| Diabetes with complications | 2.97 (2.67-3.27) | <0.001 |
| Paraplegia or hemiplegia | 8.93 (8.35-9.52) | <0.001 |
| Renal disease | 1.75 (1.57-1.94) | <0.001 |
| Cancer | 4.95 (4.56-5.34) | <0.001 |
| Metastatic cancer | 6.68 (6.14-7.21) | <0.001 |
| AIDS | 5.09 (0.85-9.33) | 0.019 |
| Pulmonary embolism | 6.67 (5.74-7.59) | <0.001 |
| Sepsis/SIRS | 12.04 (11.67-12.41) | <0.001 |
| POI Delirium | 7.52 (7.27-7.78) | <0.001 |
| POI Stroke | 2.75 (2.15-3.34) | <0.001 |
| POI ARDS | 6.26 (5.20-7.31) | <0.001 |
| POI ALI | -4.31 (-5.07- -3.55) | <0.001 |
| POI AKI | 4.16 (3.93-4.39) | <0.001 |

Myocardial infarction was omitted because of collinearity.

POI Delirium - Perioperative delirium; POI Stroke - Perioperative stroke; POI ARDS - Perioperative acute respiratory distress syndrome; POI ALI - Perioperative acute liver injury; POI AKI - Perioperative acute kidney injury
