# Supplementary material for: Chronic obstructive pulmonary disease affects outcome in surgical patients with perioperative organ injury: a retrospective cohort study in Germany
Source: Respir Res. 2024 Jun 20;25:251. doi: 10.1186/s12931-024-02882-3 (PMC11191349; doi:10.1186/s12931-024-02882-3)
Supplement: Supplementary file 15 — Supplementary Material 15 [file 12931_2024_2882_MOESM15_ESM.docx]

Additional File 15. Risk-Adjusted associations of **Hospital length of stay** from multivariable regression analysis models analysing the impact of COPD in 39,723 hospitalized surgical patients with perioperative acute respiratory distress syndrome.

|  | Coefficient (95% CI) | P- value |
| --- | --- | --- |
| COPD | 1.35 (0.51-2.20) | 0.002 |
| Age | -0.22 (-0.25- -0.20) | <0.001 |
| Female | 0.66 (0.02-1.32) | 0.043 |
| Emergency hospital admission | -2.60 (-3.22- -1.98) | <0.001 |
| *Charlson comorbidity score items* | | |
| Myocardial infarction | -0.94 (-2.54- 0.65) | 0.245 |
| Chronic heart failure | 2.05 (1.39-2.72) | <0.001 |
| Peripheral vascular disease | 0.87 (-0.01-1.75) | 0.054 |
| Cerebrovascular disease | 0.60 (-0.60-1.79) | 0.330 |
| Dementia | -2.47 (-4.35- -0.69) | <0.001 |
| Rheumatic disease | -4.28 (-6.02- -2.54) | 0.005 |
| Peptic ulcer disease | 9.64 (8.01-11.27) | <0.001 |
| Mild liver disease | 1.64 (0.44-2.84) | 0.007 |
| Moderate to severe liver disease | 1.05 (-0.65-2.76) | 0.227 |
| Diabetes without complications | 3.09 (2.31-3.86) | <0.001 |
| Diabetes with complications | 1.90 (0.57-3.23) | 0.005 |
| Paraplegia or hemiplegia | 13.95 (12.65-15.26) | <0.001 |
| Renal disease | 4.48 (3.61-5.35) | <0.001 |
| Cancer | 5.12 (4.14-6.10) | <0.001 |
| Metastatic cancer | 2.06 (0.83-3.30) | 0.001 |
| AIDS | 5.29 (1.16-9.43) | 0.012 |
| Pulmonary embolism | 2.90 (1.57-4.24) | <0.001 |
| Sepsis/SIRS | 7.99 (7.39-8.60) | <0.001 |
| POI Delirium | 14.47 (13.68-15.25) | <0.001 |
| POI Stroke | 2.74 (0.90-4.59) | 0.004 |
| POI AMI | -0.42 (-2.29-1.44) | 0.656 |
| POI ALI | -4.35 (-5.31- -3.38) | <0.001 |
| POI AKI | -0.10 (-0.76-0.55) | 0.754 |

POI Delirium - Perioperative delirium; POI Stroke - Perioperative stroke; POI AMI - Perioperative acute myocardial infarction; POI ALI - Perioperative acute liver injury; POI AKI - Perioperative acute kidney injury
