# Supplementary material for: Chronic obstructive pulmonary disease affects outcome in surgical patients with perioperative organ injury: a retrospective cohort study in Germany
Source: Respir Res. 2024 Jun 20;25:251. doi: 10.1186/s12931-024-02882-3 (PMC11191349; doi:10.1186/s12931-024-02882-3)
Supplement: Supplementary file 16 — Supplementary Material 16 [file 12931_2024_2882_MOESM16_ESM.docx]

Additional File 16. Risk-Adjusted associations of **Hospital length of stay** from multivariable regression analysis models analysing the impact of COPD in 87,485 hospitalized surgical patients with perioperative acute liver injury.

|  | Coefficient (95% CI) | P- value |
| --- | --- | --- |
| COPD | 1.25 (0.67-1.83) | <0.001 |
| Age | -0.27 (-0.28- -0.26) | <0.001 |
| Female | 0.31 (-0.01-0.63) | 0.056 |
| Emergency hospital admission | -1.41 (-1.73- -1.10) | <0.001 |
| *Charlson comorbidity score items* | | |
| Myocardial infarction | -0.96 (-1.87- -0.06) | 0.037 |
| Chronic heart failure | 4.29 (3.86-4.73) | <0.001 |
| Peripheral vascular disease | 0.56 (0.09-1.02) | 0.020 |
| Cerebrovascular disease | 1.23 (0.34-2.11) | 0.007 |
| Dementia | -1.66 (-2.40- -0.93) | <0.001 |
| Rheumatic disease | 3.42 (2.22-4.62) | <0.001 |
| Peptic ulcer disease | 5.56 (4.79-6.33) | <0.001 |
| Mild liver disease | 0.00 (-0.45- 0.46) | 0.990 |
| Moderate to severe liver disease | 2.82 (2.29-3.35) | <0.001 |
| Diabetes without complications | 1.69 (1.29-2.08) | <0.001 |
| Diabetes with complications | 1.34 (0.66-2.03) | <0.001 |
| Paraplegia or hemiplegia | 9.78 (8.58-10.98) | <0.001 |
| Renal disease | 3.70 (3.26-4.14) | <0.001 |
| Cancer | 5.67 (5.22-6.12) | <0.001 |
| Metastatic cancer | 3.19 (2.81-3.57) | <0.001 |
| AIDS | 11.10 (6.48-15.71) | <0.001 |
| Pulmonary embolism | 4.72 (3.48-5.97) | <0.001 |
| Sepsis/SIRS | 9.29 (8.88-9.71) | <0.001 |
| POI Delirium | 17.78 (17.05-18.52) | <0.001 |
| POI Stroke | 3.31 (1.70-4.93) | <0.001 |
| POI AMI | -1.20 (-2.37- -0.04) | 0.043 |
| POI ARDS | 3.09 (2.17-4.00) | <0.001 |
| POI AKI | 1.40 (1.01-1.78) | <0.001 |

POI Delirium - Perioperative delirium; POI Stroke - Perioperative stroke; POI AMI - Perioperative acute myocardial infarction; POI ARDS - Perioperative acute respiratory distress syndrome; POI AKI - Perioperative acute kidney injury
