# Supplementary material for: Chronic obstructive pulmonary disease affects outcome in surgical patients with perioperative organ injury: a retrospective cohort study in Germany
Source: Respir Res. 2024 Jun 20;25:251. doi: 10.1186/s12931-024-02882-3 (PMC11191349; doi:10.1186/s12931-024-02882-3)
Supplement: Supplementary file 17 — Supplementary Material 17 [file 12931_2024_2882_MOESM17_ESM.docx]

Additional File 17. Risk-Adjusted associations of **Hospital length of stay** from multivariable regression analysis models analysing the impact of COPD in 840,424 hospitalized surgical patients with perioperative acute kidney injury.

|  | Coefficient (95% CI) | P- value |
| --- | --- | --- |
| COPD | 1.87 (1.72-2.01) | <0.001 |
| Age | -0.22 (-0.23- -0.22) | <0.001 |
| Female | 1.59 (1.50-1.69) | <0.001 |
| Emergency hospital admission | -0.92 (-1.01- -0.82) | <0.001 |
| *Charlson comorbidity score items* | | |
| Myocardial infarction | -0.94 (-1.14- -0.74) | <0.001 |
| Chronic heart failure | 4.58 (4.48-4.68) | <0.001 |
| Peripheral vascular disease | 3.01 (2.88-3.14) | <0.001 |
| Cerebrovascular disease | -0.01 (-0.22-0.20) | 0.938 |
| Dementia | -2.26 (-2.40- -2.12) | <0.001 |
| Rheumatic disease | 3.36 (2.98-3.74) | <0.001 |
| Peptic ulcer disease | 3.97 (3.74-4.20) | <0.001 |
| Mild liver disease | 1.53 (1.30-1.75) | <0.001 |
| Moderate to severe liver disease | 2.89 (2.59-3.18) | <0.001 |
| Diabetes without complications | 1.05 (0.93-1.17) | <0.001 |
| Diabetes with complications | 1.59 (1.45-1.74) | <0.001 |
| Paraplegia or hemiplegia | 6.52 (6.23-6.81) | <0.001 |
| Renal disease | 1.50 (1.40-1.60) | <0.001 |
| Cancer | 2.57 (2.43-2.72) | <0.001 |
| Metastatic cancer | 2.51 (2.36-2.67) | <0.001 |
| AIDS | 9.29 (6.79-11.78) | <0.001 |
| Pulmonary embolism | 5.75 (5.31-6.18) | <0.001 |
| Sepsis/SIRS | 10.19 (10.06-10.33) | <0.001 |
| POI Delirium | 10.78 (10.62-10.94) | <0.001 |
| POI Stroke | 3.23 (2.83-3.62) | <0.001 |
| POI AMI | 0.75 (0.46-1.03) | <0.001 |
| POI ARDS | 4.95 (4.53-5.36) | <0.001 |
| POI ALI | -2.95 (-3.22- -2.68) | <0.001 |

POI Delirium - Perioperative delirium; POI Stroke - Perioperative stroke; POI AMI - Perioperative acute myocardial infarction; POI ARDS - Perioperative acute respiratory distress syndrome; POI ALI - Perioperative acute liver injury
