# Supplementary material for: Chronic obstructive pulmonary disease affects outcome in surgical patients with perioperative organ injury: a retrospective cohort study in Germany
Source: Respir Res. 2024 Jun 20;25:251. doi: 10.1186/s12931-024-02882-3 (PMC11191349; doi:10.1186/s12931-024-02882-3)
Supplement: Supplementary file 18 — Supplementary Material 18 [file 12931_2024_2882_MOESM18_ESM.docx]

Additional File 18. Risk-Adjusted associations of **Perioperative ventilation time** from multivariable regression analysis models analysing the impact of COPD in 490,710 hospitalized surgical patients with any perioperative organ injury.

|  | Coefficient (95% CI) | P- value |
| --- | --- | --- |
| COPD | 107.47 (104.01-110.93) | <0.001 |
| Age | -3.05 (-3.12- -2.96) | <0.001 |
| Female | -22.11 (-24.35- -19.87) | <0.001 |
| Emergency hospital admission | -15.83 (-18.05- -13.60) | <0.001 |
| *Charlson comorbidity score items* | | |
| Myocardial infarction | -34.53 (-38.08- -31.98) | <0.001 |
| Chronic heart failure | 25.21 (22.87-27.54) | <0.001 |
| Peripheral vascular disease | -23.71 (-26.34- -21.05) | <0.001 |
| Cerebrovascular disease | 33.53 (30.19-36.87) | <0.001 |
| Dementia | -49.35 (-53.52- -45.17) | <0.001 |
| Rheumatic disease | 6.34 (-3.24- 15.92) | 0.195 |
| Peptic ulcer disease | 47.01 (41.52-52.49) | <0.001 |
| Mild liver disease | -2.68 (-7.59-2.22) | 0.284 |
| Moderate to severe liver disease | -23.39 (-30.18- -16.60) | <0.001 |
| Diabetes without complications | 25.44 (22.76-28.11) | <0.001 |
| Diabetes with complications | 6.04 (1.95-10.12) | <0.001 |
| Paraplegia or hemiplegia | 84.58 (80.29-88.86) | <0.001 |
| Renal disease | 0.97 (-1.70-3.65) | 0.476 |
| Cancer | -24.52 (-28.26- -20.78) | <0.001 |
| Metastatic cancer | -68.87 (-73.01- -64.74) | <0.001 |
| AIDS | 109.13 (72.69-145.56) | <0.001 |
| Pulmonary embolism | 74.23 (67.20-81.27) | <0.001 |
| Sepsis/SIRS | 206.81 (204.40-209.23) | <0.001 |
