# Supplementary material for: Chronic obstructive pulmonary disease affects outcome in surgical patients with perioperative organ injury: a retrospective cohort study in Germany
Source: Respir Res. 2024 Jun 20;25:251. doi: 10.1186/s12931-024-02882-3 (PMC11191349; doi:10.1186/s12931-024-02882-3)
Supplement: Supplementary file 19 — Supplementary Material 19 [file 12931_2024_2882_MOESM19_ESM.docx]

Additional File 19. Risk-Adjusted associations of **Perioperative ventilation time** from multivariable regression analysis models analysing the impact of COPD in 185,968 hospitalized surgical patients with perioperative delirium.

|  | Coefficient (95% CI) | P- value |
| --- | --- | --- |
| COPD | 143.45 (137.67-149.22) | <0.001 |
| Age | -2.65 (-2.80- -2.50) | <0.001 |
| Female | -11.34 (-15.35- -7.32) | <0.001 |
| Emergency hospital admission | -25.51 (-29.26- -21.76) | <0.001 |
| *Charlson comorbidity score items* | | |
| Myocardial infarction | -23.79 (-32.00- -15.59) | <0.001 |
| Chronic heart failure | 17.90 (13.92-21.88) | <0.001 |
| Peripheral vascular disease | -27.58 (-32.01- -23.15) | <0.001 |
| Cerebrovascular disease | -1.26 (-7.36-4.84) | 0.686 |
| Dementia | -52.71 (-58.61- -46.80) | <0.001 |
| Rheumatic disease | 12.36 (-4.51- 29.23) | 0.151 |
| Peptic ulcer disease | 49.72 (40.03-59.42) | <0.001 |
| Mild liver disease | 5.18 (-4.00-14.37) | 0.269 |
| Moderate to severe liver disease | 25.10 (20.52-29.68) | 0.101 |
| Diabetes without complications | 27.40 (22.75-32.05) | <0.001 |
| Diabetes with complications | 5.78 (-1.49-13.05) | 0.119 |
| Paraplegia or hemiplegia | 95.85 (88.20-103.49) | <0.001 |
| Renal disease | 4.13 (-0.55-8.81) | 0.084 |
| Cancer | -9.55 (-15.77- -3.34) | 0.003 |
| Metastatic cancer | -44.80 (-51.81- -37.78) | <0.001 |
| AIDS | 66.86 (5.66-128.05) | 0.032 |
| Pulmonary embolism | 83.12 (70.06-96.17) | <0.001 |
| Sepsis/SIRS | 212.86 (208.40-217.31) | <0.001 |
| POI Stroke | 38.53 (29.01-48.05) | <0.001 |
| POI AMI | 17.85 (8.35-27.36) | <0.001 |
| POI ARDS | 255.30 (243.65-266.94) | <0.001 |
| POI ALI | 52.21 (38.83-65.60) | <0.001 |
| POI AKI | 66.86 (62.64-71.08) | <0.001 |

POI Stroke - Perioperative stroke; POI AMI - Perioperative acute myocardial infarction; POI ARDS - Perioperative acute respiratory distress syndrome; POI ALI - Perioperative acute liver injury; POI AKI - Perioperative acute kidney injury
