# Supplementary material for: Chronic obstructive pulmonary disease affects outcome in surgical patients with perioperative organ injury: a retrospective cohort study in Germany
Source: Respir Res. 2024 Jun 20;25:251. doi: 10.1186/s12931-024-02882-3 (PMC11191349; doi:10.1186/s12931-024-02882-3)
Supplement: Supplementary file 20 — Supplementary Material 20 [file 12931_2024_2882_MOESM20_ESM.docx]

Additional File 20. Risk-Adjusted associations of **Perioperative ventilation time** from multivariable regression analysis models analysing the impact of COPD in 62,541 hospitalized surgical patients with perioperative stroke.

|  | Coefficient (95% CI) | P- value |
| --- | --- | --- |
| COPD | 74.04 (62.03-86.05) | <0.001 |
| Age | -2.52 (-2.75- -2.29) | <0.001 |
| Female | -17.99 (-24.09- -11.89) | <0.001 |
| Emergency hospital admission | -30.30 (-36.48- -24.11) | <0.001 |
| *Charlson comorbidity score items* | | |
| Myocardial infarction | -16.29 (-31.74- -0.85) | 0.039 |
| Chronic heart failure | 15.43 (8.12-22.74) | <0.001 |
| Peripheral vascular disease | -32.85 (-40.64- -25.07) | <0.001 |
| Dementia | -69.80 (-84.12- -55.48) | <0.001 |
| Rheumatic disease | -13.82 (-46.43-18.79) | 0.406 |
| Peptic ulcer disease | 39.07 (19.63-58.51) | <0.001 |
| Mild liver disease | 22.64 (4.19-41.09) | 0.016 |
| Moderate to severe liver disease | 41.35 (0.31-82.40) | 0.048 |
| Diabetes without complications | 37.15 (29.78-44.52) | <0.001 |
| Diabetes with complications | 28.10 (13.98-42.21) | <0.001 |
| Paraplegia or hemiplegia | 15.33 (9.28-21.39) | <0.001 |
| Renal disease | 12.17 (3.00-21.34) | 0.009 |
| Cancer | -48.57- (-63.09- -34.06) | <0.001 |
| Metastatic cancer | -94.31 (-112.42- -76.19) | <0.001 |
| AIDS | 15.56 (-89.47- 120.58) | 0.772 |
| Pulmonary embolism | 45.36 (29.15-61.58) | <0.001 |
| Sepsis/SIRS | 191.23 (182.87-199.59) | <0.001 |
| POI Delirium | 5.30 (-3.09-13.69) | 0.216 |
| POI AMI | 18.21 (0.37-36.05) | 0.045 |
| POI ARDS | 185.38 (166.30-204.45) | <0.001 |
| POI ALI | 1.61 (-19.07-22.28) | 0.879 |
| POI AKI | 36.21 (28.17-44.26) | <0.001 |

Cerebrovascular disease was omitted because of collinearity.

POI Delirium- Perioperative delirium; POI AMI - Perioperative acute myocardial infarction; POI ARDS - Perioperative acute respiratory distress syndrome; POI ALI - Perioperative acute liver injury; POI AKI - Perioperative acute kidney injury
