# Supplementary material for: Chronic obstructive pulmonary disease affects outcome in surgical patients with perioperative organ injury: a retrospective cohort study in Germany
Source: Respir Res. 2024 Jun 20;25:251. doi: 10.1186/s12931-024-02882-3 (PMC11191349; doi:10.1186/s12931-024-02882-3)
Supplement: Supplementary file 21 — Supplementary Material 21 [file 12931_2024_2882_MOESM21_ESM.docx]

Additional File 21. Risk-Adjusted associations of **Perioperative ventilation time** from multivariable regression analysis models analysing the impact of COPD in 80,800 hospitalized surgical patients with perioperative acute myocardial infarction.

|  | Coefficient (95% CI) | P- value |
| --- | --- | --- |
| COPD | 71.27 (63.57-86.13) | <0.001 |
| Age | -2.33 (-2.54- -2.13) | <0.001 |
| Female | -9.00 (-13.97- -4.04) | <0.001 |
| Emergency hospital admission | 30.04 (25.53-34.54) | <0.001 |
| *Charlson comorbidity score items* | | |
| Chronic heart failure | 10.25 (5.67-14.83) | <0.001 |
| Peripheral vascular disease | -7.26 (12.70- -1.81) | 0.009 |
| Cerebrovascular disease | 7.24 (-0.58-15.06) | 0.070 |
| Dementia | -33.58 (-45.61- -21.55) | <0.001 |
| Rheumatic disease | 1.25 (-21.34-23.83) | 0.914 |
| Peptic ulcer disease | 54.38 (39.28-69.48) | <0.001 |
| Mild liver disease | 23.60 (7.89-39.31) | 0.003 |
| Moderate to severe liver disease | 1.84 (-30.18-33.87) | 0.910 |
| Diabetes without complications | 5.78 (0.68-10.88) | 0.026 |
| Diabetes with complications | -14.64 (-22.90- -6.38) | 0.001 |
| Paraplegia or hemiplegia | 100.26 (88.53-111.99) | <0.001 |
| Renal disease | -0.16 (-5.74-5.42) | 0.954 |
| Cancer | -7.82 (-19.81-4.17) | 0.201 |
| Metastatic cancer | -45.13 (-61.07- -29.19) | <0.001 |
| AIDS | -10.00 (-99.77-68.78) | 0.804 |
| Pulmonary embolism | 80.65 (58.77-102.53) | <0.001 |
| Sepsis/SIRS | 214.46 (207.80-221.13) | <0.001 |
| POI Delirium | 42.62 (36.90-48.34) | <0.001 |
| POI Stroke | 70.90 (58.03-83.77) | <0.001 |
| POI ARDS | 206.75 (189.44-224.06) | <0.001 |
| POI ALI | -8.45 (-21.59-4.68) | 0.207 |
| POI AKI | 80.95 (75.77-68.13) | <0.001 |

Myocardial infarction was omitted because of collinearity.

POI Delirium- Perioperative delirium; POI Stroke - Perioperative stroke; POI ARDS - Perioperative acute respiratory distress syndrome; POI ALI - Perioperative acute liver injury; POI AKI - Perioperative acute kidney injury
