# Supplementary material for: Chronic obstructive pulmonary disease affects outcome in surgical patients with perioperative organ injury: a retrospective cohort study in Germany
Source: Respir Res. 2024 Jun 20;25:251. doi: 10.1186/s12931-024-02882-3 (PMC11191349; doi:10.1186/s12931-024-02882-3)
Supplement: Supplementary file 22 — Supplementary Material 22 [file 12931_2024_2882_MOESM22_ESM.docx]

Additional File 22. Risk-Adjusted associations of **Perioperative ventilation time** from multivariable regression analysis models analysing the impact of COPD in 38,652 hospitalized surgical patients with perioperative acute respiratory distress syndrome.

|  | Coefficient (95% CI) | P- value |
| --- | --- | --- |
| COPD | 85.89 (71.03-100.75) | <0.001 |
| Age | -2.23 (-2.57- -1.89) | <0.001 |
| Female | -10.88 (-20.97- -0.79) | 0.035 |
| Emergency hospital admission | -29.84 (-39.53- -20.15) | <0.001 |
| *Charlson comorbidity score items* | | |
| Myocardial infarction | -14.78 (-42.98-13.42) | 0.304 |
| Chronic heart failure | 41.73 (31.14-52.33) | <0.001 |
| Peripheral vascular disease | -20.24 (-33.88- -6.59) | 0.004 |
| Cerebrovascular disease | 45.96 (26.65-65.28) | <0.001 |
| Dementia | -85.50 (-117.47- -53.53) | <0.001 |
| Rheumatic disease | 38.64 (0.49-76.80) | 0.047 |
| Peptic ulcer disease | 108.21 (82.91-133.52) | <0.001 |
| Mild liver disease | 7.17 (-11.81-26.14) | 0.459 |
| Moderate to severe liver disease | -29.05 (-54.83- -3.27) | 0.027 |
| Diabetes without complications | 45.52 (33.38-57.67) | <0.001 |
| Diabetes with complications | 12.02 (-9.48-33.52) | 0.273 |
| Paraplegia or hemiplegia | 117.17 (98.03-136.31) | <0.001 |
| Renal disease | 35.14 (21.25-49.03) | <0.001 |
| Cancer | -21.44 (-36.31- -6.58) | 0.005 |
| Metastatic cancer | -65.55 (-85.17- -45.94) | <0.001 |
| AIDS | 153.93 (77.49-230.37) | <0.001 |
| Pulmonary embolism | 80.42 (58.76-102.07) | <0.001 |
| Sepsis/SIRS | 160.44 (151.24-169.65) | <0.001 |
| POI Delirium | 156.32 (143.80-168.84) | <0.001 |
| POI Stroke | 17.14 (-8.39-42.66) | 0.188 |
| POI AMI | 35.70 (3.17-68.22) | 0.031 |
| POI ALI | -29.89 (-45.67- -14.11) | <0.001 |
| POI AKI | 39.12 (29.08-49.16) | <0.001 |

POI Delirium- Perioperative delirium; POI Stroke - Perioperative stroke; POI AMI - Perioperative acute myocardial infarction; POI ALI - Perioperative acute liver injury; POI AKI - Perioperative acute kidney injury
