# Supplementary material for: Chronic obstructive pulmonary disease affects outcome in surgical patients with perioperative organ injury: a retrospective cohort study in Germany
Source: Respir Res. 2024 Jun 20;25:251. doi: 10.1186/s12931-024-02882-3 (PMC11191349; doi:10.1186/s12931-024-02882-3)
Supplement: Supplementary file 23 — Supplementary Material 23 [file 12931_2024_2882_MOESM23_ESM.docx]

Additional File 23. Risk-Adjusted associations of **Perioperative ventilation time** from multivariable regression analysis models analysing the impact of COPD in 40,507 hospitalized surgical patients with perioperative acute liver injury.

|  | Coefficient (95% CI) | P- value |
| --- | --- | --- |
| COPD | 82.43 (68.66-96.19) | <0.001 |
| Age | -4.05 (-4.38- -3.71) | <0.001 |
| Female | -20.96 (-29.16- -12.76) | <0.001 |
| Emergency hospital admission | -20.44 (-28.64- -12.24) | <0.001 |
| *Charlson comorbidity score items* | | |
| Myocardial infarction | -28.19 (-47.70- -8.67) | 0.005 |
| Chronic heart failure | 41.44 (32.51-50.37) | <0.001 |
| Peripheral vascular disease | -17.53 (-27.50- -7.57) | 0.001 |
| Cerebrovascular disease | 48.95 (31.75-66.14) | <0.001 |
| Dementia | -51.76 (-74.44- -29.08) | <0.001 |
| Rheumatic disease | 24.58 (-8.36-57.52) | 0.144 |
| Peptic ulcer disease | 82.53 (64.33-100.72) | <0.001 |
| Mild liver disease | -20.57 (-32.51- -8.63) | 0.001 |
| Moderate to severe liver disease | -33.60 (-47.12- -20.08) | <0.001 |
| Diabetes without complications | 28.48 (18.19-38.78) | <0.001 |
| Diabetes with complications | -13.41 (-29.21-2.39) | 0.096 |
| Paraplegia or hemiplegia | 93.14 (73.07-113.20) | <0.001 |
| Renal disease | 16.19 (6.10-26.27) | 0.002 |
| Cancer | -35.94 (-47.34- -24.55) | <0.001 |
| Metastatic cancer | -81.64 (-93.66- -69.63) | <0.001 |
| AIDS | 32.56 (-52.31-117.44) | 0.452 |
| Pulmonary embolism | 41.00 (19.62-62.38) | <0.001 |
| Sepsis/SIRS | 146.38 (138.90-153.86) | <0.001 |
| POI Delirium | 163.21 (149.63-176.78) | <0.001 |
| POI Stroke | 45.22 (19.88-70.55) | <0.001 |
| POI AMI | 43.02 (20.34-65.69) | <0.001 |
| POI ARDS | 164.16 (149.30-179.03) | <0.001 |
| POI AKI | -11.95 (-21.89- -2.01) | 0.018 |

POI Delirium- Perioperative delirium; POI Stroke - Perioperative stroke; POI AMI - Perioperative acute myocardial infarction; POI ARDS - Perioperative acute respiratory distress syndrome; POI AKI - Perioperative acute kidney injury
