# Supplementary material for: Chronic obstructive pulmonary disease affects outcome in surgical patients with perioperative organ injury: a retrospective cohort study in Germany
Source: Respir Res. 2024 Jun 20;25:251. doi: 10.1186/s12931-024-02882-3 (PMC11191349; doi:10.1186/s12931-024-02882-3)
Supplement: Supplementary file 24 — Supplementary Material 24 [file 12931_2024_2882_MOESM24_ESM.docx]

Additional File 24. Risk-Adjusted associations of **Perioperative ventilation time** from multivariable regression analysis models analysing the impact of COPD in 282,658 hospitalized surgical patients with perioperative acute kidney injury.

|  | Coefficient (95% CI) | P- value |
| --- | --- | --- |
| COPD | 98.69 (94.12-103.26) | <0.001 |
| Age | -3.26 (-3.39- -3.13) | <0.001 |
| Female | -22.28 (-25.41- -19.15) | <0.001 |
| Emergency hospital admission | -19.41 (-22.53- -16.28) | <0.001 |
| *Charlson comorbidity score items* | | |
| Myocardial infarction | -30.02 (-36.68- -23.36) | <0.001 |
| Chronic heart failure | 28.23 (25.04-31.42) | <0.001 |
| Peripheral vascular disease | -20.13 (-23.76- -16.49) | <0.001 |
| Cerebrovascular disease | 26.98 (20.94-33.02) | <0.001 |
| Dementia | --56.43 (-62.25- -50.60) | <0.001 |
| Rheumatic disease | 4.98 (-7.77-17.12) | 0.444 |
| Peptic ulcer disease | 48.40 (41.70-55.09) | <0.001 |
| Mild liver disease | -8.83 (-14.81- -2.84) | 0.004 |
| Moderate to severe liver disease | -26.66 (-34.26- -19.05) | <0.001 |
| Diabetes without complications | 28.12 (24.40-31.84) | <0.001 |
| Diabetes with complications | 2.00 (-3.08-7.08) | 0.441 |
| Paraplegia or hemiplegia | 87.39 (80.29-94.49) | <0.001 |
| Renal disease | -1.01 (-4.49-2.48) | 0.572 |
| Cancer | -28.86 (-33.79- -23.49) | <0.001 |
| Metastatic cancer | -76.92 (-82.25- -71.59) | <0.001 |
| AIDS | 85.34 (36.11-134.58) | 0.001 |
| Pulmonary embolism | 60.83 (51.61-70.04) | <0.001 |
| Sepsis/SIRS | 176.27 (173.27-179.27) | <0.001 |
| POI Delirium | 81.00 (77.09-84.91) | <0.001 |
| POI Stroke | 46.66 (38.16-55.16) | <0.001 |
| POI AMI | 47.06 (39.24-54.88) | <0.001 |
| POI ARDS | 186.83 (180.04-193.62) | <0.001 |
| POI ALI | -25.33 (-30.24- -20.42) | <0.001 |

POI Delirium- Perioperative delirium; POI Stroke - Perioperative stroke; POI AMI - Perioperative acute myocardial infarction; POI ARDS - Perioperative acute respiratory distress syndrome; POI ALI - Perioperative acute liver injury
